# The actomyosin cortex controls t-tubule remodeling in skeletal muscle

**DOI:** 10.1126/sciadv.aeb3209

**Published:** 2026-09-02

**Authors:** Ana Raquel Pereira, Silvia Di Francescantonio, Ana da Rosa Soares, Tianyang Liu, Filomena A. Carvalho, Josie Liane Ferreira, Graciano Leal, Inês Faleiro, Naoko Kogata, Beatrice Labella, Teresinha Evangelista, Nuno C. Santos, Michael Way, Carolyn A. Moores, Edgar R. Gomes

**Affiliations:** ^1^ GIMM–Gulbenkian Institute for Molecular Medicine, Avenida Prof. Egas Moniz, 1649-028 Lisboa, Portugal.; ^2^ Faculdade de Medicina, Universidade de Lisboa, Av. Prof. Egas Moniz, 1649-028 Lisboa, Portugal.; ^3^ iNOVA4Health, NOVA Medical School|Faculdade de Ciências Médicas, NMS|FCM, Universidade NOVA de Lisboa, Lisbon, Portugal.; ^4^ Institute of Structural and Molecular Biology, Birkbeck College, London WC1E 7HX, UK.; ^5^ Institute of Structural and Molecular Biology, Division of Biosciences, University College London, London WC1E 6BT, UK.; ^6^ Cellular Signalling and Cytoskeletal Function Laboratory, The Francis Crick Institute, 1 Midland Road, London NW1 1AT, UK.; ^7^ Myology Institute, Groupe Hospitalier Universitaire Pitié-Salpêtrière, Paris, France.; ^8^ Functional Unit of Neuromuscular Pathology, Department of Neuropathology, Groupe Hospitalier Universitaire Pitié-Salpêtrière, APHP Sorbonne University, Paris, France.; ^9^ Sorbonne University, Myology Research Center, UMRS 974, Paris, France.; ^10^ Department of Infectious Disease, Imperial College, London SW7 2AZ, UK.

## Abstract

A network of plasma membrane invaginations called t-tubules plays an essential role in controlling calcium release from the endoplasmic reticulum at the triads during muscle contraction. Although the importance of t-tubules for muscle physiology is well established, and abnormalities are found in muscle disorders, the mechanisms that mediate t-tubule growth are unknown. We show that the actomyosin cortex beneath the plasma membrane, regulated by Arp2/3 complexes containing Arpc5, acts as a gatekeeper for the membrane availability during t-tubule growth. Enlarged t-tubules are formed upon disruption of Arpc5, impairing the synchronization between plasma membrane depolarization and calcium release. Knockout of Arpc5 in mouse skeletal muscle results in impaired locomotion and posture. Furthermore, we show that human triadopathy patients and Arpc5 knockout mice accumulate enlarged t-tubules. We propose that cortex-dependent membrane availability affects muscle function, offering a potential pathophysiological mechanism for muscle disorders.

## INTRODUCTION

The contractile properties of skeletal muscle are essential for body movement, posture, and breathing. These vital functions are affected by multiple disorders and decline with age (*1*). Sarcomeres are the contractile units of muscle cells and contract synchronously upon calcium release from the endoplasmic reticulum (ER), also known as sarcoplasmic reticulum. Muscle contraction is initiated at the neuromuscular junction by local depolarization of the plasma membrane, which propagates throughout the muscle cell (myofiber) surface (*2*). Since myofibers are the longest and thickest cells in the human body, the depolarization signal is propagated throughout their entire volume via plasma membrane invaginations called transverse tubules (t-tubules) that surround sarcomeres (*3*–*5*). These t-tubules form membrane contact sites with two adjacent ER compartments, creating a structure termed a triad. Depolarization of the t-tubule membrane triggers calcium release from the ER at the triads, a phenomenon termed excitation-contraction coupling (EC-coupling). Disruptions in triad assembly and morphology result in EC-coupling abnormalities, characteristic of triadopathies and other skeletal muscle disorders (*6*).

During mammalian development, a network of t-tubules emerges in myofibers in the embryo and becomes organized transversely only after birth (*7*). T-tubule biogenesis is initiated from plasma membrane invaginations mediated by amphiphysin 2 (Bin1), a BAR domain protein, and caveolin-3-containing caveolae (*8*). Endosomal membrane traffic has been implicated in t-tubule network formation and maintenance (*3*, *9*). In adults, t-tubules have a surface area approximately seven times that of the myofiber plasma membrane; however, it remains unclear how t-tubules grow (*10*).

The plasma membrane is tethered to the actomyosin cortex by membrane-to-cortex attachment proteins such as ezrin, radixin, and moesin (ERM) family, that affect membrane remodeling (*11*–*13*). The actomyosin cortex consists of both linear and branched actin filaments, the latter of which is nucleated by the Arp2/3 complex composed of seven evolutionarily conserved subunits (*14*, *15*). Notably, three Arp2/3 subunits are encoded by more than one gene in mammals, yielding Arp2/3 complexes with different properties (*16*–*19*). This diversity generates Arp2/3 complexes that appear to be tailored for specific cellular functions, including cell migration, viral transport, or T cell activation (*16*, *20*–*22*). In skeletal muscle, we previously demonstrated that Arp2/3 complexes containing Arpc5L drive nuclei positioning to the periphery of myofiber by promoting desmin-dependent myofibril crosslinking to generate centripetal forces on the nucleus (*23*). In contrast, Arp2/3 complexes containing Arpc5 play an essential role in triad organization by an unknown mechanism. Here, we investigated how Arpc5-containing Arp2/3 complexes are involved in t-tubule and triad organization and their impact on skeletal muscle function and health.

## RESULTS

### Inhibition of the Arp2/3 complex promotes t-tubule growth

**Fig. 1.**
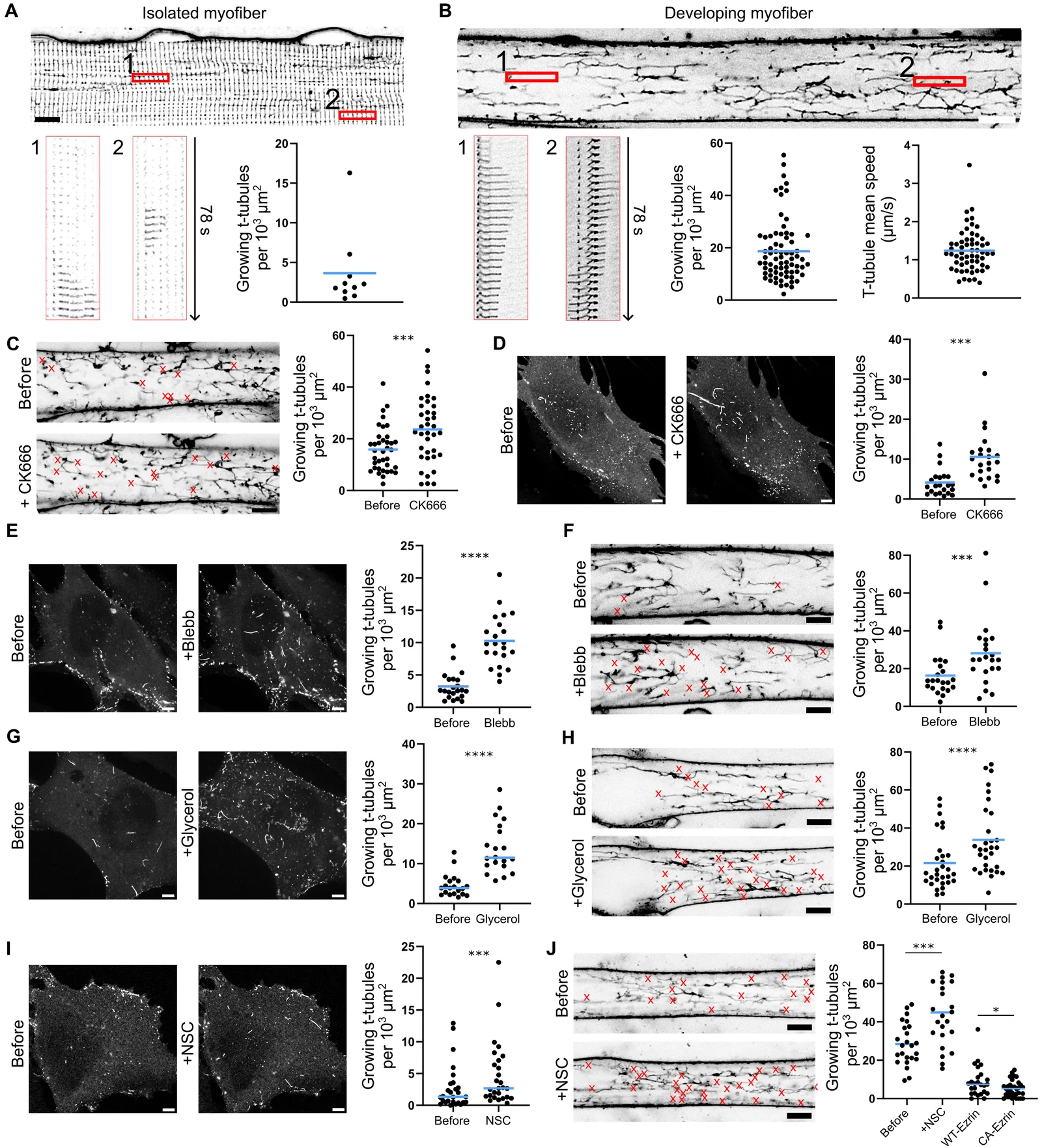
Actomyosin cortex limits t-tubule growth. (**A**) Time-lapse snapshot of a mature isolated myofiber stained with CellMask to label t-tubules and imaged every 3 s for 2 min. Kymographs from regions of interest (ROIs) 1 and 2 show representative dynamic t-tubules. Plot shows number of growing t-tubules per cell area during 2 min (*N* = 10 fibers). (**B**) Time-lapse snapshot of an in vitro developing myofiber (differentiation day 4) stained with CellMask. Kymographs from ROIs 1 and 2 show representative dynamic t-tubules. Plots show number of growing t-tubules per cell area (*N* = 72 cells) and mean growth speed (*N* = 58 growing t-tubules). (**C**) Time-lapse snapshots of the same developing myofibers (day 4) before and after CK666 addition. Red X marks sites of t-tubule growth. Plot shows number of growing t-tubules per cell area before and after CK666 (*N* = 37 myofibers). (**D**) Time-lapse snapshots of GFP-Bin1 before and after CK666. Plot shows growing tubules per cell during 2 min (*N* = 22). (**E**) GFP-Bin1 myoblasts before and after para-aminoblebbistatin (Blebb). Plot shows growing tubules per cell area (*N* = 22). (**F**) Developing myofibers before and after Blebb. Plot shows growing t-tubules per cell area (*N* = 22). (**G**) GFP-Bin1 myoblasts before and after glycerol addition. Plot shows growing tubules per cell area (*N* = 20). (**H**) Developing myofibers before and after glycerol. Plot shows growing t-tubules per cell area (*N* = 30). (**I**) GFP-Bin1 myoblasts before and after NSC668394 (NSC). Plot shows growing tubules per cell area (*N* = 27, four experiments). (**J**) Developing myofibers before and after NSC and in cells expressing WT-Ezrin-GFP or constitutively active Ezrin-GFP (CA-Ezrin, T567D). *N* = 24 (before/after NSC), *N* = 25 (WT-Ezrin), and *N* = 33 (CA-Ezrin). Scale bars, 5 μm. Unless stated, quantifications result from three independent experiments. Blue lines indicate mean values. *P ≤ 0.05, ***P ≤ 0.001, and ****P ≤ 0.0001; paired *t* test or Wilcoxon matched-pairs signed-rank test.

To understand how the t-tubule network is established in myofibers, we first evaluated t-tubule dynamics. For this, we incubated myofibers isolated from adult mice or in vitro developing myofibers with a plasma membrane dye (CellMask) to label t-tubules and performed time-lapse microscopy (*7*). We observed that t-tubules exhibit growing and shrinking movements in both isolated myofibers and developing myofibers (Fig. 1, A and B, and movies S1 and S2). T-tubule growth was independent of t-tubule-ER contacts (fig. S1 and movie S3).

We previously found that triad organization during myofiber formation depends on the Arp2/3 complex (*23*). Building on this observation, we hypothesized that the Arp2/3 complex is required for t-tubule growth. We treated developing myofibers with the Arp2/3 inhibitor CK666, and contrary to our hypothesis, there was a significant increase in the number of growing t-tubules (Fig. 1C and movie S4). T-tubule formation is mediated by Bin1, and its overexpression is sufficient to induce plasma membrane tubulation (*8*, *24*, *25*). Stable expression of green fluorescent protein (GFP)–Bin1 in C2C12 myoblasts (GFP-Bin1 myoblasts) resulted in dynamic tubules similar to those in developing myofibers (movie S5). Moreover, treatment with CK666 also increased the number of growing tubules (Fig. 1D and movie S5). Together, these results show that Arp2/3 inhibition promotes t-tubule and membrane tubule growth.

### F-actin does not accumulate near growing tubules

Actin cytoskeleton and Arp2/3 complex can modulate membrane tubulation in multiple organelles (*26*). To investigate whether actin was enriched at growing tubules, we imaged actin filaments (F-actin) using LifeAct-mCherry or mScarlet-Utrophin on membrane tubules formed in GFP-Bin1 myoblasts. We did not observe F-actin enrichment during tubule growth (fig. S2, A to D, and movies S6 and S7). We also performed cryo–correlative light and electron microscopy (cryo-CLEM) and cryo–electron tomography (cryo-ET) of GFP-Bin1 myoblasts in regions containing tubules (figs. S2, E to I, S3, and S4). We observed unbranched F-actin forming bundles or meshes throughout the different volumes (figs. S2, H and I, and S4, A and B, and movies S8 to S10). However, analysis of the volume across different z positions revealed no distinct arrangement of actin filaments near the tubules when compared to other cytoplasmic regions (figs. S2I and S4, C to J). We observed very few actin branches around tubules (fig. S2I), and they were not more abundant when compared to other regions of the cytoplasm (fig. S4, C to J). This stands in contrast to clathrin-mediated endocytosis, where branched actin is enriched near the endocytic membrane (*27*, *28*). Overall, these data suggest that branched F-actin does not accumulate near growing t-tubules, leading us to explore contributions by the Arp2/3 network to t-tubule dynamics elsewhere in the cell.

### The actomyosin cortex limits t-tubule growth

During myofiber development, the plasma membrane must be available to form t-tubules. Plasma membrane is regulated by the underlying cell cortex consisting of an actomyosin network linked to the plasma membrane by the ERM proteins (*11*, *13*). Since cortical actin is regulated by the Arp2/3 complex, we hypothesized that perturbing the actomyosin cortex leads to increased membrane availability required for t-tubule growth (*15*, *29*, *30*). To test this hypothesis, we interfered with the actomyosin cortex by inhibiting myosin-2, using para-aminoblebbistatin (blebbistatin), and quantified the number of growing tubules before and after treatment (*31*, *32*). Blebbistatin treatment of GFP-Bin1 myoblasts or developing myofibers led to a significant increase in the number of growing tubules (Fig. 1, E and F, and movies S11 and S12). We also induced an excess of plasma membrane by a rapid reduction of cell volume with a hyperosmotic shock (*33*). Quantification of the number of growing tubules before and after osmotic shock revealed an increase in the number of growing tubules (Fig. 1, G and H, and movies S13 and S14). In addition, the interference of membrane to cortex attachment with NSC668394, an ezrin phosphorylation inhibitor (*34*), also resulted in an increase in the number of growing tubules (Fig. 1, I and J, and movies S15 and S16). Conversely, overexpression of a constitutively active form of Ezrin (CA-Ezrin) (*35*) reduces the number of growing t-tubules (Fig. 1J, graph). Cells expressing CA-Ezrin had fewer growing t-tubules when compared to those with wild-type (WT)–Ezrin (Fig. 1J, graph). Together, these findings suggest that the actomyosin cortex functions as a regulatory gatekeeper in the growth of t-tubules.

### Arpc5 limits t-tubule growth through the actomyosin cortex

**Fig. 2.**
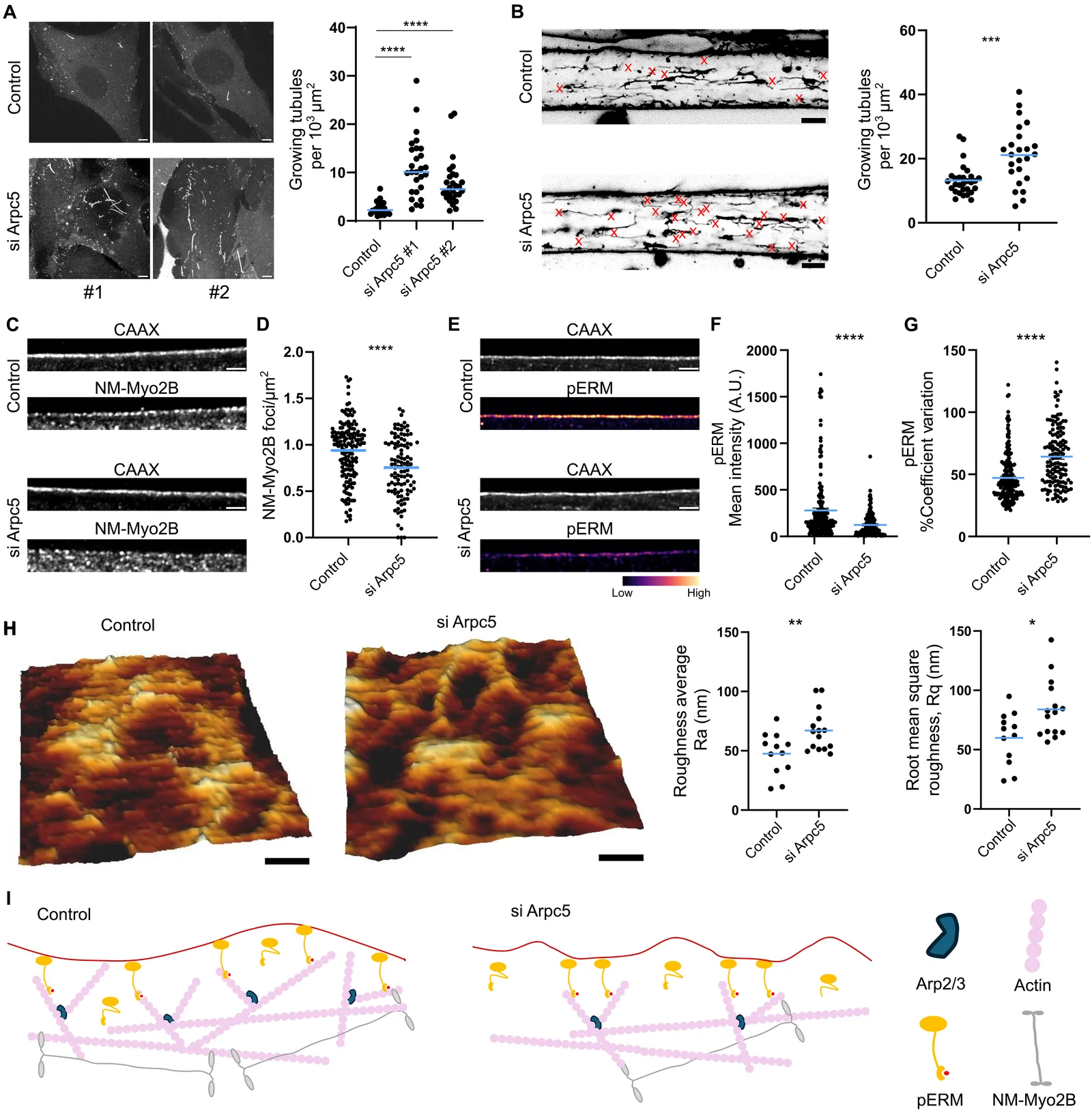
Arpc5 regulates cortical actin to limit t-tubule growth. (**A**) Time-lapse snapshots of GFP-Bin1 myoblasts transfected with scramble (control) or Arpc5 siRNA (siArpc5). Plot shows the number of growing tubules per cell area during 2 min (control *N* = 25; siArpc5#1 *N* = 27; siArpc5#2 *N* = 28). Scale bars, 5 μm. (**B**) Time-lapse snapshots of developing myofibers (day 4) with CellMask-labeled t-tubules from control or siArpc5 myofibers. Red X marks sites of t-tubule growth. Plot shows the number of growing t-tubules per cell area (control *N* = 29; siArpc5 *N* = 24). Scale bars, 5 μm. (**C**) NM-Myo2B immunostaining in CAAX-GFP myofibers from control or siArpc5 myofibers. Scale bars, 2 μm. (**D**) Quantification of NM-Myo2B foci per ROI area (square micrometers). Each point represents the average number of NM-Myo2B peaks per ROI segment area (control *N* = 164; siArpc5 *N* = 106). (**E**) pERM immunostaining in CAAX-GFP myofibers from control or siArpc5 myofibers. Scale bars, 2 μm. (**F**) pERM mean intensity quantified over consecutive 10-μm segments at the plasma membrane. Each point represents average pixel fluorescence intensity per segment length (control *N* = 188; siArpc5 *N* = 148). (**G**) Coefficient of variation of pERM signal intensity over consecutive 10-μm plasma membrane segments (control *N* = 188; siArpc5 *N* = 148). (**H**) AFM of developing myofibers. Representative quantitative imaging (QI) mode height maps shown for control and siArpc5 myofibers. Scale bars, 0.2 μm; height color scale, 200 nm. Graphs show average roughness (Ra) and root mean square roughness (Rq) for control (*N* = 12) and siArpc5 (*N* = 15) cells. (**I**) Schematic showing that Arpc5 maintains plasma membrane-to-cortex attachment and membrane flatness, gatekeeping membrane availability for t-tubule growth. Blue lines indicate mean values. Quantifications result from three independent experiments. *P ≤ 0.05, **P ≤ 0.01, ***P ≤ 0.001, and ****P ≤ 0.0001; unpaired two-tailed Student’s *t* test with Welch’s correction or Mann-Whitney test.

We showed that Arp2/3 complexes containing Arpc5 are involved in triad organization (*23*). Therefore, we hypothesized that the Arpc5 role in triad organization relates to its function in regulating cortical actin-mediated t-tubule growth. To test whether Arpc5 had a role in t-tubule growth, we performed knockdown (KD) experiments with small interfering RNA (siRNA) targeting Arpc5 in GFP-Bin1 myoblasts and developing myofibers (fig. S5). We found that depletion of Arpc5 results in significantly more growing tubules in GFP-Bin1 myoblasts and t-tubules in developing myofibers when compared to controls (Fig. 2, A and B, movies S17 and S18). Next, we evaluated the role of Arpc5 on the actomyosin cortex in developing myofibers. We found that nonmuscle myosin heavy chain II-B (NM-Myo2B) localizes as foci at the plasma membrane that were reduced upon Arpc5 depletion (Fig. 2, C and D). In addition, the levels of phosphorylated ERM (pERM) proteins were also reduced, and its distribution was more heterogeneous following Arpc5 KD (Fig. 2, E to G). Last, we assessed muscle cells’ nanoscale topology using Atomic Force Microscopy (AFM) and found that depletion of Arpc5 leads to an increase in plasma membrane roughness (Fig. 2H). These data suggest that depletion of Arpc5 affects the actomyosin cortex and the membrane to cortex attachment in developing myofibers (Fig. 2I).

### Arpc5 depletion leads to enlarged t-tubule clusters and asynchronous EC-coupling

**Fig. 3.**
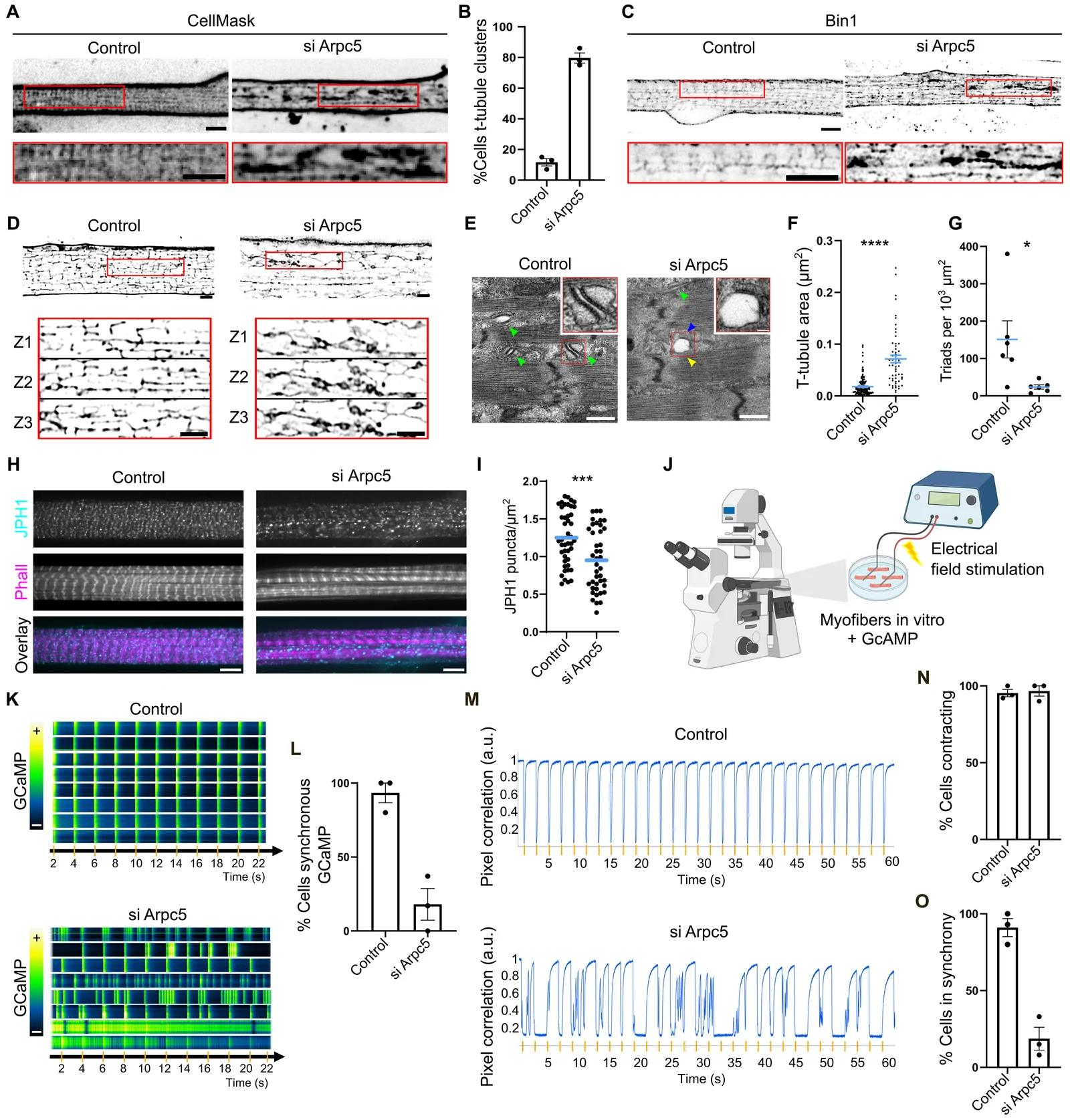
Arpc5 is involved in triad organization and synchronous EC-coupling. (**A**) Representative images of CellMask-labeled t-tubules from control or siArpc5 myofibers. Magnifications of red boxes shown below. (**B**) Percentage of myofibers with t-tubule clusters (control *N* = 42; siArpc5 *N* = 41). (**C**) Representative images of Bin1-immunostained t-tubules from control or siArpc5 myofibers. Magnifications of red boxes shown below. (**D**) Higher-resolution Airyscan reconstructions using joint deconvolution. Magnified Z-slices of red boxes shown below. Scale bars, 2 μm. (**E**) TEM of control or siArpc5 myofibers. Green arrowheads indicate triads; blue arrowhead indicates t-tubules contacting ER on one side only; yellow arrowhead indicates enlarged t-tubules. Scale bars, 500 nm; inset scale bars, 100 nm. (**F**) T-tubule area quantification (control *N* = 162; siArpc5 *N* = 56; six cells per condition; two biological replicates). (**G**) Number of triads normalized to cell area (10^3^ μm^2^). (**H**) Control or siArpc5 myofibers immunostained for JPH1 (cyan) and stained with phalloidin (magenta). (**I**) JPH1 puncta per square micrometer in control or siArpc5 myofibers (control *N* = 44; siArpc5 *N* = 41). (**J**) Schematic of EC-coupling. Created in BioRender. Francescantonio, S. (2026) https://BioRender.com/zpaxhzr. (**K**) Subcellular GCaMP fluorescence kymographs from eight myofibers per condition. Orange marks indicate electrical pulses. (**L**) Percentage of myofibers with GCaMP transients synchronized with electrical pulses (control *N* = 40; siArpc5 *N* = 62). (**M**) Representative contraction recordings from control or siArpc5 myofibers. Orange marks as (K). (**N**) Percentage of contracting myofibers (control *N* = 43; siArpc5 *N* = 36). (**O**) Percentage contracting synchronously with electrical impulses (control *N* = 41; siArpc5 *N* = 35). All experiments used day 7 myofibers. Blue lines indicate mean values; black lines indicate SEM. Scale bars, 5 μm unless stated. Quantifications from three independent experiments unless stated. *P ≤ 0.05 and ****P ≤ 0.0001; unpaired two-tailed Student’s *t* test with Welch’s correction or Mann-Whitney test.

During myofiber development, t-tubules form two-sided connections with the ER, establishing triads that become transversely organized (*3*–*5*). Since loss of Arpc5 affected triad organization (*23*), we investigated the role of Arpc5 in the t-tubule network in more differentiated myofibers (*23*, *36*). Under control conditions, we observed transversely and longitudinally aligned t-tubules which are evenly distributed throughout the myofiber (Fig. 3A). In contrast, t-tubules in Arpc5 KD myofibers formed clusters (Fig. 3, A to C). Using joint deconvolution Airyscan microscopy, we found that the clusters are composed of enlarged t-tubules (Fig. 3D). Transmission electron microscopy (TEM) of differentiated myofibers confirmed a higher proportion of enlarged t-tubules in Arpc5 KD myofibers (Fig. 3, E and F). Furthermore, we observed a reduction of triads in Arpc5 KD compared to the control both using TEM and fluorescence microscopy of Junctophilin-1 (JPH1) puncta, a marker of triads [Fig. 3, E (green arrowheads) and G to I]. Together, these results show that Arpc5 depletion affects t-tubule and triad morphology.

As triads are fundamental for EC-coupling, we assessed the impact of the loss of Arpc5 on calcium release and cell contraction in differentiated myofibers (Fig. 3J). We found that myofibers depleted of Arpc5 exhibited abnormalities in calcium transients, which were not synchronized with the electrical stimulation, when compared to WT myofibers (Fig. 3, K and L). We then performed single-myofiber contraction tracing and found that myofibers could still contract when Arpc5 was depleted (Fig. 3, M and N). However, there was a marked asynchrony between electrical stimulation and myofiber contraction (Fig. 3, M and O). These results highlight the role of Arpc5-containing Arp2/3 complexes in limiting t-tubule overgrowth and maintaining proper EC-coupling during muscle contraction.

### Arpc5 knockout affects muscle function and t-tubule morphology in vivo

**Fig. 4.**
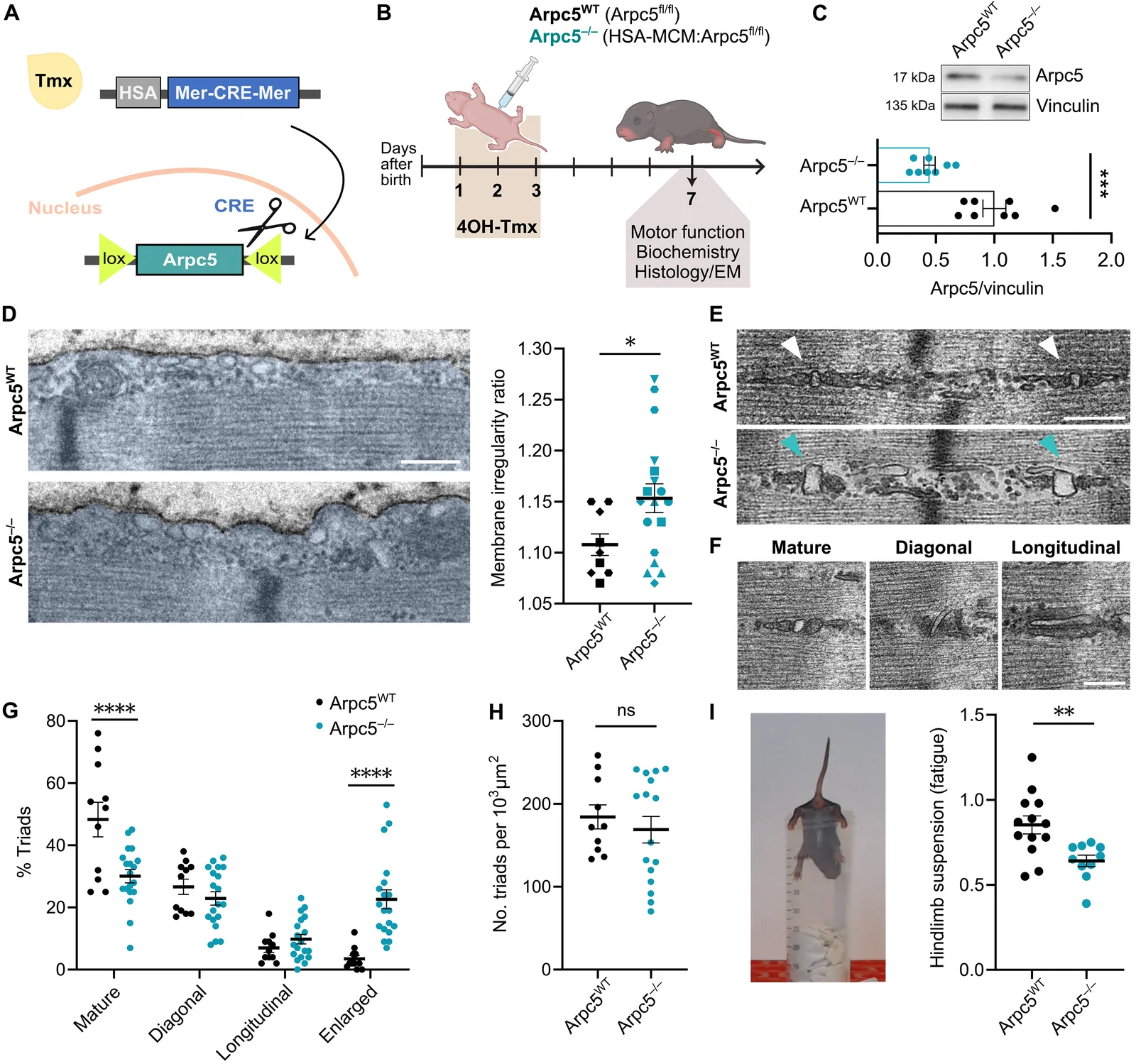
Arpc5 knockout compromises triad structure and muscle function postnatally. (**A**) Schematics of the skeletal muscle–specific inducible Arpc5 knockout strategy using the HSA-MCM Cre-lox mouse model. (**B**) Arpc5 deletion was induced by daily 4OH-tamoxifen injections from P1 to P3. At P7, locomotor performance was assessed, and hindlimb muscles were collected for TEM analysis. (A) and (B) created in BioRender. Francescantonio, S. (2026) https://BioRender.com/zpaxhzr. (**C**) Representative Western blots of Arpc5 protein levels in skeletal muscle from Arpc5^−/−^ and Arpc5^WT^ controls, normalized to vinculin and relative to controls. (**D**) Left: TEM representative images of longitudinal hindlimb muscle from control Arpc5^WT^ or Arpc5^−/−^ mice. The myofiber cytoplasm is pseudocolored in light blue. Note the darker contour line outlining the myofiber plasma membrane. Scale bar, 500 nm. Right: quantification of plasma membrane irregularity ratio in myofibers from Arpc5^WT^ and Arpc5^−/−^ mice, measuring the surface deviation from a straight line. (**E**) Representative TEM images of triads in Arpc5^WT^ and Arpc5^−/−^ muscles. White arrowheads indicate mature triads. Green arrowheads indicate enlarged t-tubules, forming an abnormal triad. Scale bar, 250 nm. (**F**) Representative examples of longitudinal, diagonal, and mature triad morphologies, illustrating progressive stages of triad maturation. Scale bar, 200 nm. (**G**) Quantifications of the percentage of diagonal, longitudinal, mature, and enlarged t-tubules at triads in myofibers. Two-way analysis of variance (ANOVA), Sidak’s multiple comparisons test; adjusted *P* value mature normal/enlarged triads <0.0001. (**H**) Quantification of total triad number normalized to cell area (10^3^ μm^2^). (**I**) Quantification of fatigue using hindlimb suspension test. Data are presented as mean ± SEM. ns, not significant; *P ≤ 0.05, **P ≤ 0.01, and ****P ≤ 0.0001 unpaired two-tailed Student’s *t* test with Welch’s correction or Mann-Whitney test based on normal distribution, unless stated otherwise. Arpc5^WT^ *N* = 13; Arpc5^−/−^ *N* = 10.

Our in vitro myofibers are differentiated up to a stage equivalent to postnatal day 1 myofibers, which have nuclei at the periphery, organized transverse triads, and a network of transverse and longitudinal t-tubules (*37*, *38*). However, the t-tubule transverse network only becomes fully established during the first weeks of postnatal development in mice (*7*, *38*). To determine whether Arpc5 has a role to further mature t-tubules and triads, we investigated mouse skeletal muscle postnatal development. As the constitutive Arpc5 knockout mouse model is embryonically lethal (*39*), we used a tamoxifen-inducible, skeletal muscle–specific Arpc5 knockout model (Arpc5^−/−^; Fig. 4A) (*40*). To induce Arpc5 knockout during postnatal development, we administered 4OH-tamoxifen immediately after birth—postnatal (P) days 1, 2, and 3—and characterized the neonatal Arpc5^−/−^ mice at P7. Arpc5 protein levels were significantly decreased when compared to controls Arpc5^fl/fl^ (Arpc5^WT^) (Fig. 4, B and C). Histological analysis of hindlimb muscle revealed an overall normal morphology and comparable myofiber size in Arpc5^−/−^ and Arpc5^WT^ mice (fig. S6). However, ultrastructural analysis by TEM showed that the plasma membrane of Arpc5^−/−^ mice was more irregular than Arpc5^WT^ (Fig. 4D). In addition, mature transverse t-tubules in triads were decreased, whereas diagonal and longitudinal t-tubules in triads did not change, suggesting that Arpc5 is also involved in t-tubule maturation that occurs postnatally (Fig. 4, E to G). We also observed an increase of enlarged t-tubules in triads (Fig. 4, E and G). Nevertheless, we found no difference in the number of triads between Arpc5^WT^ and Arpc5^−/−^ mice (Fig. 4H). Structural alterations described above were associated with functional impairments in Arpc5^−/−^ skeletal muscle physiology, as hindlimb suspension tests revealed increased fatigue and muscle weakness in Arpc5^−/−^ mice (Fig. 4I). These findings reveal the role of skeletal muscle Arpc5 in t-tubule maturation during postnatal development and indicate that t-tubule enlargement impairs muscle function.

**Fig. 5.**
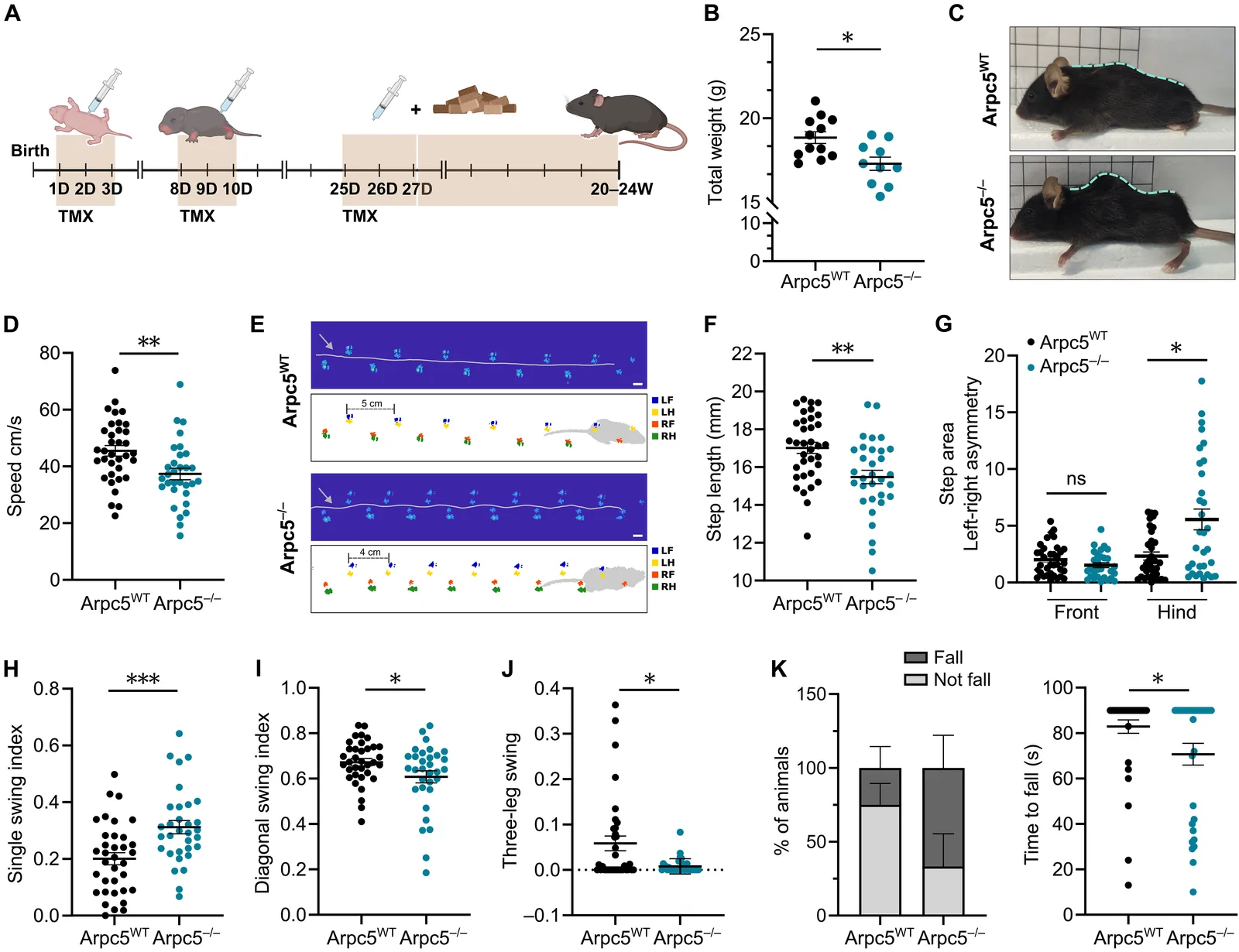
Arpc5 knockout affects locomotor function and muscle performance in adulthood. (**A**) Schematic of the inducible, skeletal muscle–specific HSA-MCM mouse model used for adult analysis. To prolong the muscle-specific Arpc5 knockout into adulthood, 4OH-tamoxifen was administered at days 1 to 3, 9 to 11, and 25 to 27, followed by a tamoxifen-enriched diet until adulthood. Muscle function was assessed, and TA and gastrocnemius muscles were collected for histological analysis. Created in BioRender. Francescantonio, S. (2026) https://BioRender.com/zpaxhzr. (**B**) Body weight of Arpc5^WT^ and Arpc5^−/−^ adult mice. (**C**) Representative images of Arpc5^WT^ and Arpc5^−/−^ mice; note the pronounced spinal deformity (kyphoscoliosis) in Arpc5^−/−^ mice. Grid spacing, 1 cm. (**D**) Quantification of walking speed in Arpc5^WT^ and Arpc5^−/−^ mice. Each dot corresponds to a single run. (**E**) Heatmaps represent a complete footprint pattern of Arpc5^WT^ and Arpc5^−/−^ mice showing pixel intensity in each step, and the horizontal line represents the body path (gray arrow). Individual feet are labeled with different colors as indicated: left fore (LF), left hind (LH), right fore (RF), and right hind (RH). Scale bars, 1 cm (120 px). (**F**) Step length of Arpc5^WT^ and Arpc5^−/−^ mice. (**G**) Left-right asymmetry index calculated on the basis of forepaw and hindpaw area normalized to total muscle weight of Arpc5^WT^ and Arpc5^−/−^ mice. Plots relative to (**H**) single, (**I**) diagonal, and (**J**) three-leg swing index in Arpc5^WT^ and Arpc5^−/−^ adult mice. (**K**) Left: percentage of Arpc5^WT^ and Arpc5^−/−^ animals that fell at least once during the wire hang. Right: wire hang performance of Arpc5^WT^ and Arpc5^−/−^ mice, shown as hang time before falling across three trials. Higher values indicate better muscular performance and endurance. Data are presented as mean ± SEM. *P ≤ 0.05, **P ≤ 0.01, and ***P ≤ 0.001 using unpaired two-tailed Student’s *t* test with Welch’s correction or Mann-Whitney test based on normal distribution. *N* ≥ 10 mice.

**Fig. 6.**
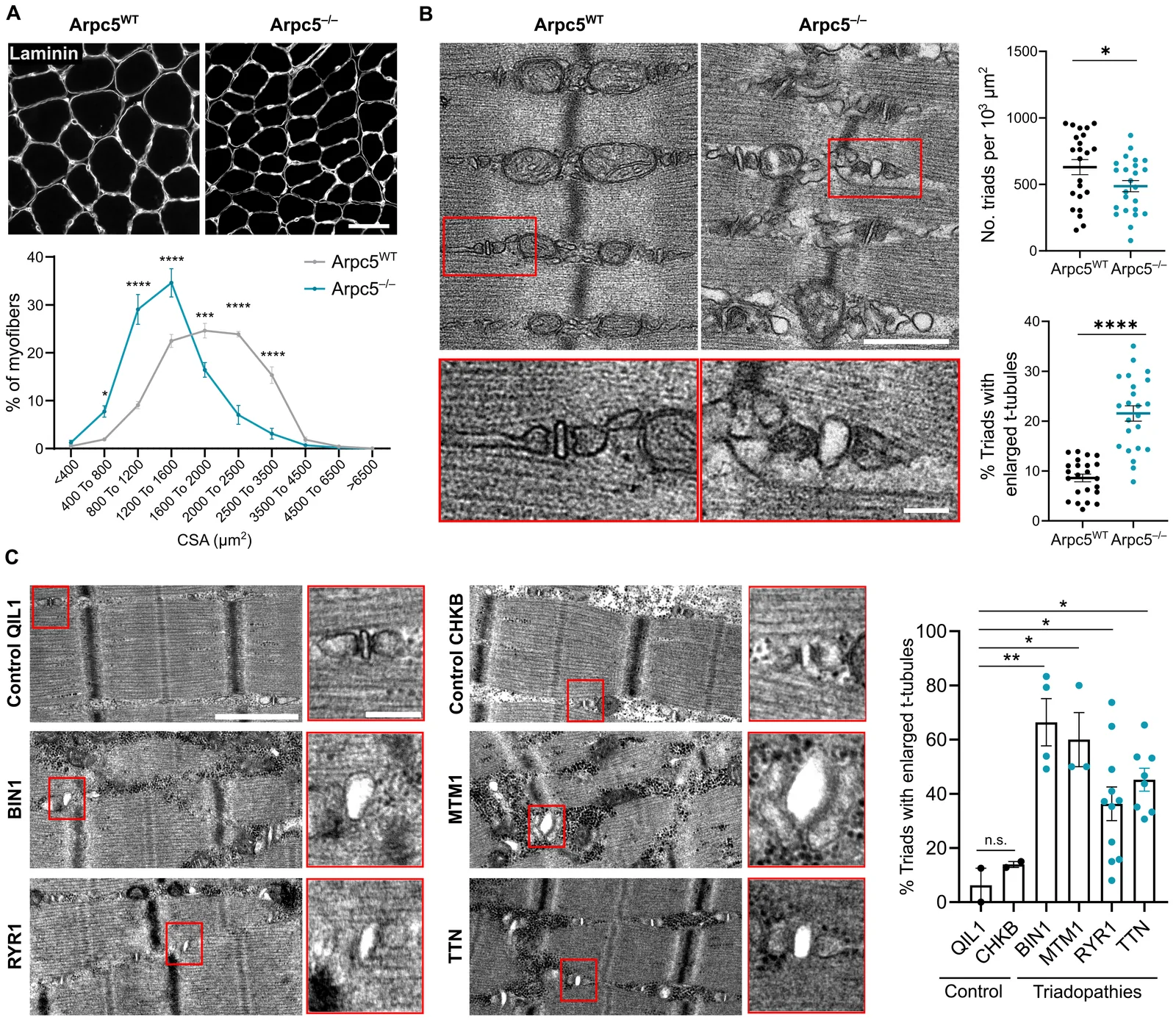
Arpc5 knockout mice and human triadopathies exhibit t-tubule enlargement. (**A**) Top: representative images of Arpc5^WT^ and Arpc5^−/−^ TA muscle cross sections stained for the basal lamina (laminin, white). Scale bar, 50 μm. Bottom: frequency distribution of myofiber CSA of adult Arpc5^WT^ and Arpc5^−/−^ mice (*N* = 4). (**B**) Left: TEM representative images of myofibers from control Arpc5^WT^ or Arpc5^−/−^ mice. Scale bar, 0.5 μm. Zoom image scale bar, 0.1 μm. Right top: quantifications of the number of triads normalized for cell area (10^3^ μm^2^) in myofibers from Arpc5^WT^ and Arpc5^−/−^ adult mice. Right bottom: quantifications of the percentage of enlarged triads in myofibers from Arpc5^WT^ and Arpc5^−/−^ adult mice (*N* = 4). Each dot corresponds to a fiber. (**C**) Left: TEM images of human patients containing mutations in QIL1 and CHKB (control, metabolic disease); BIN1, MTM1, RYR1, and TTN (triadopathy mutations). Scale bar, 1 μm. Magnifications corresponding to the red ROIs are shown as insets. Scale bar, 200 nm. Right: quantification of the percentage of triads with enlarged t-tubules. Statistical comparison was performed between QIL1 and the indicated samples. Data are presented as mean ± SEM. *P ≤ 0.05, **P ≤ 0.01, ***P ≤ 0.001, and ****P ≤ 0.0001 using unpaired two-tailed Student’s *t* test with Welch’s correction or Mann-Whitney test based on normal distribution.

To investigate the functional role of Arpc5 in skeletal muscle, we knocked out Arpc5 from birth to adulthood (Fig. 5A). Body weight of Arpc5^−/−^ mice was reduced when compared to controls (Arpc5^WT^) and presented a pronounced spinal deformity, namely, kyphoscoliosis (Fig. 5, B and C). We then evaluated locomotion performance using the MouseWalker assay in freely walking rodents (*41*, *42*). Compared with controls, Arpc5^−/−^ mice walk slower and showed reduced step length, indicating impaired locomotor performance (Fig. 5, D to F, and movie S19). The hindlimb asymmetry index was increased in Arpc5^−/−^ mice, indicating reduced left-right symmetry in hindlimb paw placement compared with control animals (Fig. 5, E and G). In addition, gait-related parameters were altered, including increased single swing index and decreased diagonal and three-leg swing indices, indicating altered interlimb coordination and support distribution (Fig. 5, H to J). Next, we investigated muscle force–related performance using a whole body wire hang test and observed that Arpc5^−/−^ mice tend to fall more and faster than the control (Fig. 5K). We also observed clear differences in hanging posture between Arpc5^−/−^ and Arpc5^WT^ (fig. S7A). We next performed histological analysis of tibialis anterior (TA) muscle, which revealed no hallmarks of damage, regeneration, or immune cell infiltration of Arpc5^−/−^ and Arpc5^WT^ tissue (fig. S7B). However, Arpc5^−/−^ muscle myofibers have a reduced cross-sectional area (CSA) and perimeter when compared to Arpc5^WT^ (Fig. 6A and fig. S7C). In addition, TEM of TA Arpc5^−/−^ adult mouse muscles showed enlarged t-tubules and fewer triads when compared to Arpc5^WT^ (Fig. 6B). Together, these results suggest a role of Arpc5 in muscle function, namely, posture and locomotion in adult mice, associated with reduction of myofiber size, enlarged t-tubules, and reduced triad number.

### Enlarged t-tubules are a common feature of human triadopathy diseases

Triadopathies encompass congenital skeletal muscle disorders in which mutations in BIN1, MTM1, RYR1, or TTN disrupt triad structure and muscle function (*6*). Abnormal t-tubule morphology, including the t-tubule enlargement we observe upon Arpc5 loss in mice, has been reported in Bin1-CNM (*43*). To determine whether t-tubule enlargement represents a shared pathological hallmark across human triadopathies, we systematically analyzed TEM images of patient muscle biopsies and quantified the proportion of triads exhibiting enlarged t-tubules. As disease controls, we examined biopsies from patients with metabolic muscle diseases (QIL1 and CHKB myopathies), in which primary triad involvement has not been described (*44*, *45*). We found that patients with mutations in BIN1, MTM1, RYR1, and TTN showed a significant increase in the proportion of triads with enlarged t-tubules compared to controls (Fig. 6C). Together with our mouse data, these observations identify t-tubule enlargement at triads as a conserved morphological hallmark of triad dysfunction.

## DISCUSSION

T-tubules and their connection with the ER to form triads are fundamental for muscle contraction and are disrupted in multiple muscle disorders (*6*). We now show that the actomyosin cortex, regulated by Arpc5-containing Arp2/3 complexes, acts as a gatekeeper for membrane availability required for t-tubule growth. This is essential for ensuring proper myofiber development and muscle function and provides a previously unidentified pathophysiological mechanism for muscle disorders with impaired triad function.

Our observations reveal that the destabilization of the actomyosin cortex releases plasma membrane, enabling t-tubule extension. Membrane tubulation has also been observed following stretch release in adherent cells, suggesting that membrane release facilitates tubule formation (*46*). In addition, elegant experiments using giant unilamellar vesicles have demonstrated that membrane release alone, induced by a reduction in vesicle volume, can drive inward tubulation, mimicking t-tubule formation without the need for external forces (*47*). We did not observe enrichment of actin or accumulation of branched actin near growing t-tubules. While we cannot exclude the involvement of external forces, our data do not support a requirement for actin-mediated force generation in t-tubule growth. Bin1 has also been shown to induce membrane tubulation independently of actin (*48*). Bin1, along with proteins such as dynamin-2, likely contributes to t-tubule growth by stabilizing membrane curvature, in synergy with the mechanism we identify (*48*, *49*).

Cardiac and skeletal t-tubules share similar roles in muscle, but their structures are distinct. In cardiac muscle, wider t-tubules are shaped into membrane microdomains by a cardiac specific BIN1 isoform (*50*–*52*). In skeletal muscle, t-tubules are about 10 times narrower and rely on skeletal BIN1 isoforms (*3*, *8*, *24*, *53*). The absence of the microfold-forming BIN1 isoform, combined with the geometric constraints imposed by narrower t-tubule lumen, probably explains why microdomains have not been observed in skeletal t-tubules (*2*, *3*, *8*). These muscle type–specific distinctions support the view that the cortex-dependent regulation of membrane availability we describe here is a mechanism particularly well suited to the scale and growth requirements of skeletal muscle t-tubules. Therefore, we propose a model in which limiting t-tubule growth is essential for proper triad formation, synchronized EC-coupling, triads, muscle physiology, and locomotion. Excessive t-tubule growth may disrupt membrane contacts with the ER, impairing triad assembly and function.

During postnatal development, t-tubules and triads undergo spatial consolidation into a transverse orientation, and our results suggest that actomyosin cortex regulation has a role in this process (*7*, *38*). In P7 Arpc5^−/−^ muscle, t-tubule enlargement was already evident despite preserved myofiber size and unchanged triad number, indicating that abnormal t-tubule morphology is an early consequence of Arpc5 knockout. With prolonged Arpc5 loss into adulthood, this early structural defect was accompanied by reduced myofiber size, fewer triads, kyphoscoliosis, impaired gait, and reduced force-related performance. Thus, sustained disruption of Arpc5-dependent cortical regulation may progressively compromise triad organization and muscle integrity, ultimately affecting posture and locomotion. Together, these results indicate that cortical control of membrane availability remains important beyond initial t-tubule formation, contributing to the maintenance of t-tubule morphology and skeletal muscle function.

T-tubule overgrowth and triad abnormalities have been observed in aged human muscle, and altered t-tubule morphology has been reported as a recurrent feature of several skeletal muscle diseases (*3*, *6*, *43*, *54*). Consistent with this, our systematic analysis of patient biopsies carrying mutations in triadopathy-associated genes revealed an increased proportion of triads with enlarged t-tubules. Notably, several muscle disorders display features that overlap those observed in Arpc5 knockout mice, including kyphoscoliosis, impaired muscle function, and enlarged t-tubules (*55*–*58*). Moreover, patients with ARPC5 loss-of-function variants also present scoliosis and neuromuscular impairment, further supporting the relevance of this pathway in human disease (*59*). Thus, the mechanism identified here may define a common vulnerability across distinct muscle disorders, although further studies will be required to determine the role of Arpc5 in these diseases and to assess whether cortical control of membrane availability represents a potential therapeutic target.

## MATERIALS AND METHODS

### T-tubule labeling in mature myofibers

All procedures using animals were approved by the Institutional Ethics Committee and followed the guidelines of the Portuguese National Research Council Guide [Direção Geral da Alimentação e Veterinaria (DGAV)]. Ex vivo isolated single myofibers from extensor digitorum longus (EDL) muscles were generated using the protocol previously described (*36*). EDL muscle was explanted from 8- to 12-week-old male or female C57BL/6J mice and then digested in Dulbecco’s modified Eagle’s medium (DMEM; Invitrogen) containing 0.2% (w/v) type I collagenase (Sigma-Aldrich) for 2 hours at 37°C. Mechanical dissociation of fibers was performed using a thin Pasteur pipette in a transilluminating-fluorescent stereomicroscope. Single fibers were collected and plated in ibidi 35-mm dishes precoated with 1% (v/v) Matrigel in Iscove’s modified Dulbecco’s media (IMDM; Invitrogen) with GlutaMAX. After plating, cells were covered with an upper coating of ice-cold 50% (v/v) Matrigel coating in IMDM with GlutaMAX. Plated myofibers were maintained in 10% (v/v) horse serum (Thermo Fisher Scientific) plus IMDM with GlutaMAX for the duration of the experiment. To visualize t-tubule dynamics, plated myofibers were labeled for 30 min with CellMask Orange (1 μM, Invitrogen).

### In vitro myofibers

All animal procedures were approved by the institutional ethics committee and followed the guidelines of the National Research Council Guide for the care and use of laboratory animals. In vitro myofibers were differentiated as previously described (*60*) from primary myoblasts isolated from 5- to 7-day-old mixed-gender C57BL/6 mice. TA, EDL, gastrocnemius, and quadriceps muscles were dissected and kept in ice-cold phosphate-buffered saline (PBS). Muscles were digested mechanically using surgical scissors and incubated for 90 min at 37°C with agitation in collagenase type V (Sigma-Aldrich) and dispase II (Roche) in DPBS. Digestion was stopped by mixing 10% (v/v) of fetal bovine serum (FBS) plus 1% (v/v) of penicillin-streptomycin IMDM with GlutaMAX. The cells suspension was centrifuged for 5 min at 75*g* to remove debris followed by a second centrifugation for 5 min at 350*g*. After centrifugation, cells were resuspended in the same medium and preplated for 4 hours to allow for fibroblast adherence. The cells remaining in suspension were collected, centrifuged for 5 min at 350*g*, and plated at a density of 220,000 cells/ml in 20% (v/v) FBS plus IMDM with GlutaMAX in ibidi 35-mm dishes precoated with 1% (v/v) Matrigel in IMDM with GlutaMAX. After 3 days of proliferation, medium was replaced by 10% (v/v) horse serum IMDM with GlutaMAX supplemented with recombinant agrin (100 ng/ml) to induce differentiation. An ice-cold upper coating of 50% (v/v) Matrigel in 10% (v/v) horse serum IMDM with GlutaMAX was added the following day, and fresh medium was added. Experiments were performed either at 4 to 5 or 7 to 8 days after differentiation.

### Mice

All the animal procedures were reviewed and approved by the institutional ethics committee GIMM ORBEA and followed the guidelines of the Portuguese National Research Council Guide (DGAV project license nos. 022871/2016 and 004560/2022) for the care and use of laboratory animals. All mice used in these studies were housed at the DGAV-accredited rodent facility of Gulbenkian Institute for Molecular Medicine, in individually ventilated cages within a specific and opportunistic pathogen–free facility, on a standard 12/12-hour light cycle.

Skeletal muscle–specific Arpc5 knockout strain mice were developed by breeding Arpc5 floxed mice (*39*) with mice expressing Cre recombinase (MerCreMer or MCM) double fusion protein under the human α-skeletal actin (HSA) promoter (*40*). The HSA promoter drives the expression of a tamoxifen-inducible Cre recombinase specifically in skeletal muscle. Upon tamoxifen administration, the Cre recombinase will be translocated in the nucleus where it will excise the Arpc5 floxed gene only in myofibers (Fig. 4A). For each experiment, cre-negative littermates were used as controls (Arpc5^WT^). Both males and females were used without distinction. 4-Hydroxytamoxifen (Sigma-Aldrich, H6278) was injected intraperitoneally (1.3 mg/ml in CornOil) at P1, P2, and P3. At P7 to P8, motor function was evaluated, and/or animals were euthanized by decapitation. Hindlimb muscle was dissected for biochemistry or histological analysis and electron microscopy (EM) processing. For adult studies, 4-hydroxytamoxifen (1.3 mg/ml in CornOil) was administered again by intraperitoneal injection on P9 to P10 (50 μl) and P25 to P27 (100 μl), followed by a tamoxifen-enriched diet (SNIFF, A155T70400, 400 mg/kg) until adulthood (20 to 24 weeks). Behavioral assays were performed between weeks 20 and 24. Hindlimb muscles (gastrocnemius and TA) were then dissected for biochemical, histological, or EM analysis.

### Motor function evaluation

#### Neonates

To evaluate motor function postnatally, we used a neonate appropriate hindlimb suspension test to measure muscle fatigue (*61*).

Briefly, pups were placed gently facing down into a 50-ml conical tube, with their hind legs hung over the rim. This was repeated three times, with ∼30 s of resting time among each trial. Fatigue was assessed by calculating the ratio between the time to fall in the final trial and the initial trial. This fatigue index (final time/initial time) reflects the decline in endurance capacity over repeated tests. A lower value indicates a greater degree of fatigue, as a reduced ability to sustain suspension over time.

#### Adults

*Whole body hanging test*. Adult mice (20 to 24 weeks) were placed on a 30 cm–by–50 cm wire grid (0.5-cm^2^ mesh openings) that was inverted and held 1 m above a soft landing surface, as previously described (*62*). Each mouse underwent three trials with a 10-min intertrial interval. The outcome measure was the latency to fall (maximum 90 s per trial). We recorded the hang time across three trials per animal.

*MouseWalker assay*. Locomotor performance was assessed using the MouseWalker system (*41*, *42*), an open-source hardware and software platform designed to provide a comprehensive and quantitative description of kinematic features in freely walking rodents. Mice were placed on a walkway equipped with a frustrated total internal reflection setup, and high-speed video was recorded as the animals voluntarily walked. The recorded videos were processed using the MouseWalker tracking and analysis toolbox to extract kinematic and coordination metrics. To assess interlimb coordination and bilateral symmetry, left-right asymmetry indices for the forelimbs and hindlimbs were calculated as the difference between the left and right paw area values. All gait parameters were analyzed using custom postquantification scripts, as previously described (*42*, *63*) or GraphPad Prism (v. 8.4.3. of GraphPad Software Inc.) and compared between genotypes using an unpaired two-tailed Student’s *t* test with Welch’s correction or Mann-Whitney test.

### siRNAs, plasmids, and transfections

To image F-actin, GFP-Bin1 myoblasts were transfected with 1-μg plasmid/ibidi 35-mm dish of LifeAct-mCherry (*64*) or mScarlet-I-UtrCH (gift from D. Gadella, Addgene plasmid #98823; https://addgene.org/98823; RRID:Addgene_98823) 1 day after plating, using Lipofectamine 2000 (Thermo Fisher Scientific), according to the manufacturer’s protocol. For Arpc5 depletion, GFP-Bin1 myoblasts at 30% confluency were transfected with Thermo Fisher Scientific Silencer Select siRNAs for Arpc5. Two different siRNAs for Arpc5 were used: #1 (ID #s85623) and #2 (ID #s85621) at 20 nM/ibidi 35-mm dish using Lipofectamine RNAiMAX (Thermo Fisher Scientific). Silencer Select Scramble (ID #4390843) was used as a negative control. Cells were imaged by a vtiSIM microscope on the next day (see below).

Triadin-GFP (Trisk95-GFP (*65*), WT-Ezrin (Ezrin-GFP, gift from G. Charras laboratory), or CA-Ezrin (EzrinT567D-GFP, gift from G. Charras laboratory) plasmids were transfected to in vitro myofibers 3 days after plating (differentiation day 0) with 1 μg of DNA per ibidi 35-mm dish of the appropriate plasmid using Lipofectamine 2000 (Thermo Fisher Scientific), according to the manufacturer’s protocol. To deplete Arpc5 in myofibers, Thermo Fisher Scientific Silencer Select siRNAs for Arpc5 #1 (ID #s85623) was transfected 3 days after plating at 20 nM/ibidi 35-mm dish using Lipofectamine RNAiMAX (Thermo Fisher Scientific). Silencer Select Scramble (ID #4390843) was used as a negative control.

### Generating the GFP-Bin1 myoblast stable cell line

The GFP-Bin1 stable cell line of C2C12 myoblasts (GFP-Bin1 myoblasts) was generated using a pLVX-puromycin vector containing Bin1 isoform 8. The lentiviral vector was constructed with VectorBuilder. The vector ID is VB220728-1365nbr, which can be used to retrieve detailed information about the vector on vectorbuilder.com. Lentiviruses were produced in human embryonic kidney (HEK) 293T cells. To generate stable cell lines expressing exogenous GFP-Bin1, C2C12 myoblasts were plated in a six-well plate. The cells with a 10% confluency were carefully covered with 0.5 ml of filtered medium containing the lentiviruses, and 0.5 ml of complete medium was added to myoblasts (1 ml total volume). The medium containing lentiviruses was left during 8 hours, and then 1 ml of complete fresh medium was added. Cells were allowed to grow until 80% of confluency. Subsequently, GFP-positive cells were sorted by FACS, allowed to expand, and the process was repeated for three rounds of sorting to achieve a homogeneous cell population. GFP-Bin1 myoblasts were cultured in DMEM with glutamine (Gibco) supplemented with 10% FBS (Gibco) and penicillin-streptomycin (100 U/ml:100 μg/ml; Gibco), at 37°C under 5% CO_2_.

### AAV1-CAG-GFP-CAAX production

The AAV1-CAG-GFP-CAAX was generated following the protocols from Addgene with minor modifications (*66*, *67*). Briefly, production began by triple transfecting the pAAV-CAG-CAAX plasmid (see Supplementary Text for plasmid map) with AAV packaging plasmid expressing Rep2 and Cap1 (#112862, Addgene) and pHelper into 15-cm plates of confluent HEK 293T cells using polyethylenimine (Polysciences Inc., catalog no. 23966-100). The cells were harvested 3 days posttransfection. The purification step was performed by iodixanol gradient ultracentrifugation, and the viral fraction was concentrated to ∼150 μl using an Amicon Ultra-15 centrifugal filter unit (Merck, catalog no. UFC910024). For measuring virus titer, we performed quantitative polymerase chain reaction (Bio-Rad CFX96 real-time polymerase chain reaction system), using primers against woodchuck hepatitis virus posttranscriptional regulatory element.

### Fluorescence microscopy

Airyscan images were acquired using a Zeiss LSM980 Airyscan2 microscope, equipped with a 63× Plan-Apochromat differential interference contrast (DIC) oil objective [numerical aperture (NA) 1.4], unless stated otherwise. Imaging of fixed cells was performed at RT, and imaging of live cells was performed at 37°C with 5% of CO_2_ using the ZEN 3.8 blue edition. To measure t-tubule growth, images were recorded every 3 s. Airyscan reconstruction was performed using automatic filter settings. To visualize the t-tubule network of in vitro differentiated myofibers at higher resolution, Airyscan images were processed using joint deconvolution in ZEN blue, which is an accelerated joint Richardson-Lucy algorithm (Zeiss).

For instant structured illumination microscopy imaging, the vtiSIM module (Visitech) equipped on a Nikon Tie microscope with a 100× AC Oil CFI SR HP Apo TIRF objective and a Photometrics Prime BSI Express Scientific sCMOS camera were used. Time-lapse imaging was performed at 37°C with 5% CO_2_.

To visualize t-tubule dynamics in developing myofibers, the membrane lipid dye CellMask Orange (1 μM, Invitrogen) was added to 4-day differentiated myofibers 5 to 10 min before imaging. At this stage of differentiation, t-tubules are starting to form and have a longitudinal orientation. T-tubule and/or Triadin-GFP dynamics were recorded by time-lapse imaging using Airyscan microscopy (see above). To visualize actin dynamics near growing t-tubules, GFP-Bin1 myoblasts transfected with LifeAct-mCherry or mScarlet-I-UtrCH were imaged by vtiSIM time lapse every second for 3 min.

To visualize t-tubule network and t-tubule clusters, developed in vitro myofibers (differentiation day 7) were incubated with 1 μM CellMask Deep red (Invitrogen). Spinning disk confocal imaging was performed using a Zeiss Axio Observer microscope equipped with a Yokogawa CSU-X1 scanning unit, an Evolve 512 EMCCD camera and a 63× Plan-Apochromat DIC oil objective (NA 1.4), using the ZEN 3.2 blue edition software. Cells were kept at 37°C and 5% CO_2_. Myofibers with more than three t-tubule clusters were visually quantified using Fiji software (*68*), and the percentage of cells with t-tubule clusters was plotted using GraphPad.

### Drug treatments

Cells were treated with 100 μM CK666 (Sigma-Aldrich) for Arp2/3 inhibition. A final concentration of 125 μM of para-aminoblebbistatin (Motorpharma) was used to block myosin-II. To inhibit Ezrin phosphorylation, NSC668394 (MedChemExpress) was used at 50 μM. The number of growing tubules or t-tubules was assessed immediately after drug treatments for GFP-Bin1 myoblasts and after 5 min for myofibers. T-tubule and tubule dynamics were recorded by time-lapse imaging using Airyscan microscopy. GFP-Bin1 myoblasts treated with para-aminoblebbistatin or glycerol were recorded by time-lapse imaging using vtiSIM microscopy.

### Glycerol treatment

Myoblasts and developing myofibers were exposed to 225 and 45 mM glycerol, respectively, to induce hyperosmotic shock. The number of growing t-tubules was performed 3 min after glycerol treatment for GFP-Bin1 myoblasts and after 5 min for myofibers. Myofiber t-tubule dynamics were recorded by time-lapse imaging using Airyscan microscopy. GFP-Bin1 myoblasts t-tubule dynamics were recorded by time-lapse imaging using vtiSIM microscopy.

### Immunostainings

Developing myofibers (differentiation day 4) or differentiated myofibers (differentiation day 7) were fixed in 4% paraformaldehyde in PBS for 10 min at room temperature and permeabilized with 0.5% Triton X-100 for 5 min. Cells were blocked in a solution containing 10% goat serum, 5% bovine serum albumin (BSA), and VisUBlock Mouse on Mouse Blocking Reagent (R&D Systems, VB001). Primary and secondary antibodies were diluted in 0.1% saponin. Cells were incubated with primary antibodies overnight at 4°C and washed three times for 10 min with PBS. Cells were incubated with secondary antibodies for 1 hour at room temperature and washed three times for 10 min with PBS. Cells were mounted with Fluoromount-G (Invitrogen).

For JPH1 immunostaining in differentiated myofibers (day 7), we used an anti-JPH1 antibody (1:200 dilution, Invitrogen, 40-5100) and an anti-rabbit Alexa Fluor 488 secondary antibody (1:400, Life Technologies). Phalloidin conjugated with an Alexa Fluor 647 (1:200, Life Technologies) was used to stain filamentous actin. Myofibers were imaged using the vtiSIM microscope (VisiTech).

For Bin1 immunostaining in differentiated myofibers (day 7), we used an anti-Bin1 antibody (1:200 dilution, Sigma-Aldrich, 05-449) and an anti-mouse Alexa Fluor 555 (1:400 dilution, Life Technologies). Myofibers were imaged using the LSM980 microscope with Airyscan with a Plan-Apochromat 63×/1.40 Oil DIC M27, oil immersion objective.

For the measurement of myofibers’ CSA, the TA muscle from adult mice or hindlimb muscle from pups was wrapped in tragacanth gum and frozen in liquid nitrogen–cooled isopentane. Transverse 6- to 10-μm cryosections were prepared from the median part of the muscles with a cryostat, and after thawing to RT, heat-induced epitope retrieval was performed in the cryosections using a PT module (A80400012, Thermo Fisher Scientific, New Hampshire, USA) at 95°C for 20 min in EnVision FLEX Target Retrieval Solution Low pH (Dako, Agilent Technologies, Denmark). After 1 hour of washing in PBS-T (PBS containing 0.1% Tween 20), the tissue was permeabilized, and nonspecific sites were blocked with 5% BSA and 0.1% Triton X-100 in PBS for 2 hours at RT. AffiniPure Fab Fragment Goat Anti-Mouse (Jackson ImmunoResearch) was used. Sections were incubated with a laminin primary antibody (1:200, Thermo Fisher Scientific, MA1-06100) O/N at 4°C in 2% BSA, 0.1% Triton X-100, and 5% goat serum in PBS. After washing in PBS-T, tissue was incubated with anti-mouse Alexa Fluor 488 or 647 secondary antibodies (1:400, Life Technologies) for 2 hours at RT in 2% BSA, 0.1% Triton X-100, and 5% goat serum in PBS. After another round of extensive washes with PBS-T and subsequent drying at RT, cryosections were mounted in Fluoromount G (Thermo Fisher Scientific). Hematoxylin and eosin staining (Merck, HT110132-1L) was performed on muscle transverse cryosections according to the manufacturer’s instructions. Tiles of the whole sections or specific positions were acquired using a Zeiss Axioscan 7 or Zeiss Cell Observer microscope.

### p-ERM and NM-Myo2B localization at the plasma membrane

To label the plasma membrane, in vitro primary myofibers were infected on day 2 of differentiation with the AAV1-CAG-GFP-CAAX (1.535 × 10^7^ genome copies to a 35-mm dish containing myofibers at ∼70 to 80% confluency). The virus was washed on the next day, with differentiation media containing agrin (100 ng/ml). At day 4 of differentiation, cells were fixed and stained as described above.

To label pERM, we used an anti–phospho-Ezrin (Thr^567^)/radixin (Thr^564^)/moesin (Thr^558^) (1:200 dilution, Cell Signaling Technology, #3141) and an anti-rabbit Alexa Fluor 555 secondary antibody (1:400, Life Technologies). To label anti-nonmuscle myosin heavy chain II-B (NM-Myo2B), cells were incubated with an NM-Myo2B antibody (1:200 dilution, BioLegend) and an anti-rabbit Alexa Fluor 555 secondary antibody (1:400, Life Technologies). Cells were also costained with an anti-GFP antibody (1:200 dilution, Roche, #11814460001) and an anti-mouse Alexa Fluor 488 secondary antibody (1:400, Life Technologies). Cells were imaged by Airyscan using the LSM980 microscope with a 40× LD C-Apochromat, NA 1.1, water immersion objective.

To quantify the number of foci of NM-Myo2B, using Fiji, we drew rectangle regions of interest (ROIs) with a constant width of 0.8 μm along the CAAX-GFP channel. The NM-Myo2B fluorescence intensity signal over that ROI was then plotted, and the number of peaks (corresponding to NM-Myo2B foci) was measured with the Find Peaks BAR tool (*69*) using a mean amplitude of 100. The number of peaks in each ROI was divided by the respective area of the ROI, and these values were plotted as the number of NM-Myo2B foci per ROI area (square micrometers).

To quantify pERM at the plasma membrane, we used the CAAX-GFP signal to identify the plasma membrane and drew a line of 10-μm length by 10 pixels width on top of the signal and extracted the single-pixel intensity values of pERM. To calculate the pERM intensity signal, we averaged the single-pixel intensity over the 10-μm line. To measure the pERM coefficient of variation, we calculated the SD of the single-pixel intensity over the 10-μm line divided by the mean of fluorescence.

### Atomic force microscopy

AFM imaging of developing myofibers was performed using a NanoWizard IV system (Bruker-JPK Instruments, Berlin, Germany) mounted atop an Axiovert 200 inverted optical microscope (Carl Zeiss, Jena, Germany). To avoid cell contraction during acquisition, developing myofibers (differentiated for 4 days) were imaged in tyrode buffer [140 mM NaCl, 5 mM KCl, 2 mM MgCl_2_, and 10 mM Hepes (pH 7.2)]. For AFM imaging, oxidized and sharpened silicon probes with a nominal tip radius of ∼6 nm, a resonance frequency of 60 kHz, and a spring constant of 3 N/m were used. Scanning was performed at an optimized speed of 0.3 Hz using a minimal applied force to avoid damaging the sample. Images were acquired at a resolution of 512 × 512 pixels.

Plasma membrane surface roughness measurements were conducted on live cells following 4 days of differentiation, using the same equipment. Experiments were performed at 25°C in Tyrode’s solution, using the quantitative imaging (QI) mode to capture height data. Nonfunctionalized qp-BioAC CB2 cantilevers (Nanosensors, Neuchâtel, Switzerland) with partial gold coating and quartz-like tips, featuring a nominal spring constant of 100 pN/nm, were used. DIC microscopy facilitated accurate positioning of the cantilever tip over both Arpc5 KD and scramble control cells. QI mode height maps of 5 μm by 5 μm were acquired with a vertical (*z*) range of 2 μm. Images were recorded at a resolution of 256 × 256 pixels with a pixel dwell time of 20 ms. During each approach, the cantilever-to-cell distance was dynamically adjusted to maintain a maximal applied force of 1 nN before tip retraction. For each experimental condition, a minimum of 12 height images were obtained, with all samples analyzed in triplicate. Image processing was performed using JPK Image Processing software (v. 8.0.144) to calculate plasma membrane average roughness (Ra) and root mean square roughness (Rq). For each height map, roughness was determined in five distinct 0.5 μm–by–0.5 μm square regions located between Z-bands. In total, surface topographies of 12 control cells and 15 Arpc5 KD cells were analyzed.

### Image analysis

All images were processed in Fiji (*68*). For cell drift correction, we used the Fiji plugin Fast4Dreg (*70*). For t-tubule growth speed measurements, manual tracking of the tip of the t-tubule during the time lapse was performed using Fiji manual tracking.

The number of growing t-tubules was manually recorded using the Fiji cell counter tool. This value was then normalized for the cell area. All quantifications were performed within 2-min time lapses (42 frames). For the cells expressing WT-Ezrin or CA-Ezrin, the number of growing t-tubules was measured in cells containing GFP-positive signal only.

To measure Triadin-GFP mean speed in developing myofibers, we used Airyscan images of Triadin-GFP time lapses acquired every 3 s for 2 min. Since Triadin-GFP localizes as distinct spots, we used the Fiji plugin Trackmate (*71*) to track Triadin-GFP spots over time in myofibers. To detect Triadin-GFP spots, we used the LoG detector with an estimated object diameter of 0.5 μm and a quality threshold of 5, 10, or 20. We then linked the spots to generate tracks, by using the LAP tracker with a max distance of 0.5 μm and no gap closing. To have enough data points to calculate speed trajectories, we only considered tracks that lasted more than 10 frames (30 s). The resulting tracked Triadin-GFP spots were then used to calculate mean speed.

To quantify actin dynamics in the vicinity of growing t-tubules, we manually selected an ROI around GFP-Bin1 growing tubules [as the ones represented in fig. S2 (A and C)], including a few time points before the GFP-Bin1 signal was observed using Fiji software. We then extracted at each time point the average of fluorescence intensity per square micrometer for GFP-Bin1, LifeAct-mCherry, or mScarlet-I-UtrCH using the Fiji software. Values were normalized for the initial corresponding average intensity, and these normalized intensity values per square micrometer over time were plotted using GraphPad.

To automatically count the number of JPH1 puncta, a maximum projection of the Z-stack images that were in focus was first performed using Fiji. Then, a rectangular ROI with an area of 175.552 μm^2^ was drawn along the myofiber length. To detect the puncta automatically, we used the LoG detector tool from the Fiji TrackMate plugin (*71*). LoG filters for puncta detection were a blob diameter of 0.5 μm and an intensity threshold of 5. To analyze the number of JPH1 puncta per square micrometer, the number of detected puncta was normalized for an ROI area and plotted. For transversal myofiber morphometric measurements, CSA was segmented using laminin staining and Cellpose (*72*).

### Western blot

Cell extracts were collected in 100 mM tris-HCl (pH 8) with 1% (w/v) SDS. Tissue was extracted from snap-frozen muscle samples and homogenized using an Omni Soft Tissue Tip Homogenizer (Biolabproducts GmbH). Protein lysates were prepared using radioimmunoprecipitation assay buffer [NaCl 150 mM, EDTA 5 mM, 1% (v/v) Triton X-100, 0.5% (v/v) NP-40, and 50 mM tris (pH 7.5)] with protease and phosphatase inhibitors (Roche). All protein quantification was performed using the BCA Protein Assay Kit (Pierce) following the manufacturer’s protocol. Samples were denatured in Laemmli buffer and heated at 95°C for 5 min. Ten to 30 μg of the total protein was loaded onto a 10% (v/v) precast polyacrylamide gel (Bio-Rad) and transferred to a nitrocellulose membrane for 75 min at 100 V. The membranes were blocked with tris-buffered saline with 0.1% (v/v) Tween 20 containing 5% skim milk, followed by incubation with primary antibodies, overnight at 4°C. Membranes were washed and incubated with horseradish peroxidase (HRP)–conjugated secondary antibodies for 1 hour at room temperature and washed three times before image acquisition. Images were acquired using Amersham ImageQuant800 (Cytiva). Antibodies used were as follows: anti-Arpc5 (1:500, Santa Cruz Biotechnology, sc-166760); anti-vinculin (1:1000, Sigma-Aldrich, V9131); anti-rabbit HRP (1:5000, Thermo Fisher Scientific); and anti-mouse HRP (1:5000, Thermo Fisher Scientific). For Arpc5 protein level quantification in Fig. 4C, results are shown as fold versus the average of littermate controls Arpc5^WT^.

### Cryo-ET sample preparation

GFP-Bin1 myoblasts were grown to ∼60% confluency and then diluted to a confluency of 10%. EM grids (Quantifoil R3.5/1 Au 200 mesh) were glow-discharged using a plasma cleaner (PLASMAFLO PDC-FMG-2, Harrick Plasma) and then placed in a 35-mm MatTek dish in a flow hood. Three hundred microliters of fibronectin (5 μg/ml; Sigma-Aldrich, 341635) was added to the dish, covering the EM grids. The dish with the grids inside was then sterilized under ultraviolet light for 1.5 hours. After simultaneous coating and sterilization, fibronectin was removed from the MatTek dish. The grids were then washed twice with PBS and stored in ∼300 μl of medium [DMEM high glucose + pyruvate + l-glutamine; 10% (v/v) FBS + 1% (v/v) PenStrep].

Once the cells were ready, the medium in the grid-containing dish was removed, and diluted cells were added to the dish. The cells were seeded on the EM grids at 37°C 5% CO_2_ overnight (∼15 hours). The following day, grids were inspected under an inverted biological microscope (Medline Scientific, 3650.0000) to check the cell density and ensure grid integrity. Grids with minimally broken areas and well-distributed cells were plunge frozen using Leica GP2 with back blotting for 15 s and an additional movement of 0.5 mm at 37°C and 90% humidity.

### Correlative light and EM

Frozen grids were first loaded onto a Titan Krios microscope (Thermo Fisher Scientific) operated at an accelerating voltage of 300 kV with a K3 detector and a BioQuantum energy filter (Gatan). Grid atlas images were collected to check the ice quality and the cell density using EPU software (Thermo Fisher Scientific). After locating the spread cells, grids were transferred onto a cryo-light microscope (Leica THUNDER Imager EM Cryo CLEM). Z stack fluorescence and bright-field images were taken of the located spread cells using LAS X software at ×50 magnification. With the fluorescence images processed using THUNDER technology, individual tubular fluorescence signals were observed. The fluorescence images were saved for fluorescence microscopy and EM image correlation. The grids were then transferred back to a Titan Krios microscope (Thermo Fisher Scientific) operated at an accelerating voltage of 300 kV, with a Selectris X image filter (Thermo Fisher Scientific) and a Falcon 4i camera (Thermo Fisher Scientific). Medium magnification atlases were taken and combined on each fluorescence-labeled cell for manual correlation and locating the ROIs. Empty holes and dark features were used as markers to facilitate the manual correlation (fig. S3, B and C). After correlation, ROIs with fluorescence signals were targeted for cryo-ET tilt series data collection. One hundred fourteen tilt series were collected using TOMO5 software with a dose symmetric tilt scheme ±60° with the step size of 3° at the magnification of ×42,000, a pixel size of 3.0 Å, a dose rate of 12.6 e^−^/pixel per s, an exposure time of 2.69 s, and a total dose per micrograph of 3.66 e^−^/Å^2^.

### Tomogram reconstruction

Tomograms were reconstructed using RELION 4.1 tomography pipeline (*73*). Raw data were imported into RELION followed by motion correction with RELION’s own implementation of MotionCor2 (*74*) and CTF correction with ctffind 4.1 (*75*). Tilt images with significant contamination and grid bars were manually excluded from further data processing in a Napari-based viewer (Exclude Tilt Images job). Then, each tilt series was aligned using IMOD patch tracking with a patch size of 100 nm and a patch overlap of 50%. The aligned tilt series were used to generate tomograms with a binned pixel size of 10 Å. Tomograms were denoised using CryoCARE to improve the signal-to-noise level (*76*). CryoCARE training was performed with 1200 subvolumes per tomogram and a subvolume dimension of 72 pixel. Upon inspecting the tomograms, we found that 58 contained vesicular as opposed to tubular membrane structures (as shown in fig. S3G) and were excluded. Twenty-three of the tomograms were discarded because of the presence by nonvitreous ice and could not be used for segmentation. In addition, 27 tomograms did not correlate well with fluorescence signals because of the ambiguity of fluorescence and the lack of correlation markers, which posed challenges for manual correlation. Six tomograms displayed tubular structures at locations where fluorescence was observed. From these, the three highest quality tomograms were selected for segmentation and further analysis.

### Tomogram segmentation

Tomograms were processed using IsoNet to further improve the signal-to-noise level (*77*). The following parameters were used: CTD deconvolve snrfalloff of 0.5, mask making density_percentage of 100, std_percentage of 100, and z_crop of 0.17. Then, IsoNet-processed tomograms were cropped to remove regions lacking t-tubule structures to simplify segmentation. Segmentation was performed using the neural network–based software Dragonfly [Dragonfly 2024.1 (Computer software). Comet Technologies Canada Inc., Montreal, Canada; software available at https://dragonfly.comet.tech/]. For each tomogram, actin and membrane structures were manually annotated on 5 to 12 sub-areas across different slices of the tomogram. The segmented data were then used to train multiple deep learning models. The model segmenting the highest number of actin filaments on test slices was selected to segment the entire tomogram. The UNet++ model (2.5D, three slices, depth level of 5, and initial filter count of 32) was used for tomograms 1 and 2. In addition, a pretrained UNet model provided by the Dragonfly team (depth level of 5 and initial filter count of 64) was used for tomogram 3. The segmentations were manually cleaned and corrected in Dragonfly. The segmented actin and membrane structures were rendered into a 3D contour mesh and smoothed over 5 to 10 iterations for three-dimensional (3D) visualization in figs. S2 (H and I) and S4. Movies were created by exporting the segmented actin and membrane structures as binary files, converting these into two MRC files, and then importing them along with a contrast-inverted 3D tomogram into ChimeraX for visualization (*78*).

### Tomogram analysis

For tomogram analysis, subtomograms proximal to the t-tubules and distant from the t-tubules were selected. The subvolumes near t-tubules were constrained to regions no more than 200 nm from the t-tubule structures. Actin branches—inferred from the relative position of two connected filaments—within these boxes were identified, labeled, and counted. For control purposes, subvolumes located more than 200 nm away from t-tubules were also identified, and suspected actin branches within these control subvolumes were similarly labeled and counted. In total, 7 subvolumes were selected to determine the number of actin branches near the t-tubules, and 3 subvolumes were selected as controls.

### Single-myofiber contraction and calcium transient assay

In vitro myofibers were plated on 35-mm fluorodishes (World Precision Instruments). After 7 to 8 days of differentiation, developed myofibers were imaged using a Nikon Eclipse Ti microscope. Myofibers were kept at 37°C and CO_2_ 5% using an Okolab stage incubator throughout all experimental procedures. Electrical field stimulation using the MyoPacer system (Ionoptix) was used to induce excitation-contraction of myofibers using 10 V at 0.5-Hz frequency pulses with a duration of 20 ms.

To measure cytosolic calcium transients during electrical pulses, myofibers at differentiation days 3 to 4 were infected using the AAV9-pAAV.CAG.GCaMP6s (1 μl/ml).WPRE.SV40 [Addgene viral prep #100844-AAV9; the plasmid was a gift from D. Kim & GENIE Project (*79*)]. After overnight incubation, the media was replaced with differentiation media [IMDM with GlutaMAX supplemented with recombinant agrin (100 ng/ml)]. After 7 to 8 days of differentiation, GCaMP fluorescence during electrical field stimulation was recorded using a 60× Plan Apo DM Ph3 (NA 1.4) objective and an Andor Sona CSC-00324 camera at a 20 ms frame rate with the NIS-Elements AR 5.42.05 software. Fiji software was then used to generate subcellular GCaMP fluorescence kymographs by drawing a 475.7-μm^2^ square ROI along the myofiber that was then was maximum projected, generating GCaMP fluorescence kymographs. The number of cells that presented GCaMP transients synchronous with the electrical pulse was then quantified from these kymographs and plotted.

For single-myofiber contraction tracing, electrical field stimulation was performed as mentioned above. Individual cell contractions were recorded with a MyoCam-S3 camera (Ionoptix) and a 40× CFI Plan Apochromat Lambda D objective (NA 0.95), using the IonWizard 7.7.3.180 software. Contraction kinetics were based on CytoMotion (Ionoptix) Pixel Correlation changes relative to the reference frame taken at rest (i.e., before electrical pulses). To generate single-myofiber contraction tracings, pixel correlation (a.u.) curves were plotted using the Cytosolver Cloud (Ionoptix). Myofibers that during the electrical stimuli did not contract, contract, and present synchronous or asynchronous contraction during the electrical stimulus were then quantified and plotted.

### Transmission electron microscopy

Developed myofibers (7 days of differentiation) were fixed for 1 hour at 4°C in 0.1 M Sorensen’s phosphate buffer (pH 7.4) containing 2.5% (v/v) glutaraldehyde (Science Services EMS), 2% (v/v) formaldehyde (Science Services EMS), and 50 mM calcium chloride (Sigma-Aldrich). Samples were postfixed for 1 hour at 4°C in the dark with 0.1 M Sorensen’s phosphate buffer (pH 7.4) containing 1% (w/v) osmium tetroxide (Science Services EMS) and 0.8% (w/v) potassium ferrocyanide (Sigma-Aldrich). After five washes with distilled water, samples were stained for 1 hour at room temperature with 0.5% (w/v) uranyl acetate (Analar). Dehydration was performed using an ethanol gradient (30% 10-min room temperature, 50% 10-min room temperature, 75% overnight 4°C, 90% 10-min room temperature, and 100% three times 10 min each room temperature). Samples were embedded in Embed-812 epoxy resin (Science Services EMS) and hardened at 60°C for at least 24 hours. Ultrathin sections (70 nm) were obtained and collected in homemade formvar carbon–coated copper-palladium slot grids and counterstained with 1% (w/v) uranyl acetate in 70% methanol and Reynolds lead citrate, for 5 min each.

For muscle tissue from mice, longitudinal sections of postnatal myofibers from mouse neonates and adults were generated from hindlimb muscles. For this, after sacrifice, the skin was removed, and the whole mouse leg was incubated with fixative 0.1 M Sorensen’s phosphate buffer (pH 7.4) containing 2.5% (v/v) glutaraldehyde (Science Services EMS) and 2% (v/v) formaldehyde (Science Services EMS) for 30 min/1 hour, to preserve muscle structure. For mouse neonates, 50 mM calcium chloride (Sigma-Aldrich) was also added during fixation. After rinsing in 0.1 M cacodylate buffer, dissection was performed by cutting small longitudinal pieces (<1 mm^3^). These small pieces were incubated overnight at 4°C with fixative 0.1 M Sorensen’s phosphate buffer (pH 7.4) containing 2.5% (v/v) glutaraldehyde (Science Services EMS) and 2% (v/v) formaldehyde (Science Services EMS) with or without 50 mM calcium chloride (Sigma-Aldrich). Samples were postfixed for 2 hours at 4°C in the dark with 0.1 M Sorensen’s phosphate buffer (pH 7.4) containing 2% (w/v) osmium tetroxide (Science Services EMS) and 0.8% (w/v) potassium ferrocyanide (Sigma-Aldrich). After five washes with distilled water, samples were stained for 1 hour at room temperature with 0.5% (w/v) uranyl acetate (Analar). Dehydration was performed using an ethanol gradient (30% 10-min room temperature, 50% 10-min room temperature, 75% overnight 4°C, 90% 10-min room temperature, and 100% three times 10 min each room temperature). Samples were embedded in Embed-812 epoxy resin (Science Services EMS) and hardened at 60°C for at least 24 hours. Ultrathin sections (70 nm) were obtained and collected in homemade formvar carbon–coated copper-palladium slot grids and counterstained with 2% (w/v) uranyl acetate in 70% MetOH and Reynolds lead citrate, for 5 min each. ROIs were mapped with SerialEM software, and tiles of differentiated cells were acquired using a FEI Tecnai G2 Spirit BioTWIN (120 kV) transmission electron microscope with an Olympus-SIS Veleta charge-coupled device camera.

Stitching was performed using the IMOD blendemont function (*80*). Stitched tiles were opened in Fiji to count the number of triads (one t-tubule connected to two ER) in each tile and normalized it to the cell area. For mouse muscle, the percentage of mature triads (one transverse elongated t-tubule in contact with two adjacent ER compartments), longitudinal, diagonal, and enlarged triads was calculated on the total number of triads. To measure t-tubule area in vitro, we identified the membrane objects that had a low electron-dense lumen and that were contacting the ER (green or blue arrowheads in Fig. 3E and white arrowheads in Fig. 4). Area was measured by drawing an ROI along the t-tubule membrane, using the drawing tool from Fiji software. We quantified the plasma membrane irregularity ratio as a measure of plasma surface roughness. The irregularity ratio was calculated by dividing the length of the manually traced membrane contour by the length of a straight horizontal reference line drawn between the two ends of the membrane segment. Membrane outlines were manually traced using high-resolution TEM images in Fiji software. Values equal to one correspond to a flat membrane. Values higher than one correspond to the degree of membrane irregularity/roughness.

### Analysis of TEM from muscle patients

Muscle biopsies were performed for diagnostic purposes in accordance with French regulations. All patients provided written informed consent for the reuse of their biological samples and clinical data for research. In France, additional approval from an ethics committee is not required for the secondary research use of diagnostic samples with patient consent. The specimens were fixed in glutaraldehyde and included in epon. Digital photographs of biopsies were obtained with AxioCam HRc HIS-0043 (Zeiss, Germany). We selected TEM images acquired at magnifications ranging from ×3000 to ×7000. To investigate whether t-tubule enlargement represents a shared morphological feature across human triadopathies, we analyzed TEM images of patient samples. Images were selected on the basis of the clear identification of triad structures, acquired at magnifications ranging from ×3000 to ×7000. To increase the number of samples analyzed, we also analyzed TEM images from human patients from the Muscle Atlas database (https://muscleatlas.institut-myologie.org). For triadopathies, we analyzed 27 TEM images (two patients with mutation in BIN1, four images; five patients with mutation in RYR1 gene, nine images; six patients with TTN mutations, eight images; and two patients with MTM1 mutations, three images). As a control, we analyzed TEM muscle samples from patients with metabolic muscle diseases from Muscle Atlas, in which triad involvement has not been described (*n* = 4, one patient with QIL1 mutation, two images; and one patient with CHKB mutation, two images) (*44*, *45*). For each image, triads were manually counted and classified as containing either normal or enlarged t-tubules using Fiji (ImageJ). Enlarged t-tubules were defined on the basis of increased diameter compared to adjacent structures. For each image, the percentage of triads exhibiting enlarged t-tubules was calculated. Data were plotted and analyzed using GraphPad Prism.

### Data representation and statistics

All graphs were generated, and statistical analyzes were conducted using GraphPad Prism v8. Normality testing was carried out using the Shapiro-Wilk test, with an α level = 0.05. Details about the tests used are described in the figure legends. Figures were formatted with InkScape (version 1.2.2). Cartoons were created in InkScape using graphical elements from BioRender.

## Supplementary Materials

### The PDF file includes:

Supplementary Text Figs. S1 to S7 Legends for movies S1 to S19 References
