# Supplementary material for: The actomyosin cortex controls t-tubule remodeling in skeletal muscle
Source: Sci Adv. 2026 Sep 2;12(36):eaeb3209. doi: 10.1126/sciadv.aeb3209 (PMC13537240; doi:10.1126/sciadv.aeb3209)
Supplement: Supplementary file 1 [file sciadv.aeb3209_sm.pdf]

Science Advances
AAAS

Supplementary Materials for

The actomyosin cortex controls t-tubule remodeling in skeletal muscle

Ana Raquel Pereira *et al.*

Corresponding author: Edgar R. Gomes, edgar.gomes@gimm.pt

Sci. Adv. **12**, eaeb3209 (2026)
DOI: 10.1126/sciadv.aeb3209

The PDF file includes:

Supplementary Text
Figs. S1 to S7
Legends for movies S1 to S19
References

Other Supplementary Material for this manuscript includes the following:

Movies S1 to S19

Supplementary Text

pAAV-CAG-GFP-CAAX_VectorMap

ttgagatcctttttttctgcgcgtaatctgctgcttgcaaacaaaaaaaccaccgctaccagcggtggtttgtttgccggatcaagagctaccaa
ctctttttccgaaggtaactggcttcagcagagcgcagataccaaatactgttcttctagtgtagccgtagttaggccaccacttcaagaactct
gtagcaccgcctacatacctcgctctgctaatcctgttaccagtggctgctgccagtggcgataagtcgtgtcttaccgggttggactcaaga
cgatagttaccggataaggcgcagcggtcgggctgaacggggggttcgtgcacacagcccagcttggagcgaacgacctacaccgaac
tgagatacctacagcgtgagctatgagaaagcgccacgcttcccgaagggagaaaggcggacaggtatccggtaagcggcagggtcgg
aacaggagagcgcacgagggagcttccagggggaaacgcctggtatctttatagtcctgtcgggtttcgccacctctgacttgagcgtcgat
ttttgtgatgctcgtcaggggggcggagcctatggaaaaacgccagcaacgcggcctttttacggttcctggccttttgctggccttttgctca
catgttctttcctgcgttatcccctgattctgtggataaccgtattaccgcctttgagtgagctgataccgctcgccgcagccgaacgaccgag
cgcagcgagtcagtgagcgaggaagcggaagagcgcccaatacgcaaaccgcctctccccgcgcgttggccgattcattaatgcagctg
cgcgctcgctcgctcactgaggccgcccgggcaaagcccgggcgtcgggcgacctttggtcgcccggcctcagtgagcgagcgagcg
cgcagagagggagtggccaactccatcactaggggttccttgtagttaatgattaacccgccatgctacttatctacgtagccatgctctagg
aagatcgtaccattgacgtcaataatgacgtatgttcccatagtaacgccaatagggactttccattgacgtcaatgggtggactatttacggta
aactgcccacttggcagtacatcaagtgtatcatatgccaagtacgccccctattgacgtcaatgacggtaaatggcccgcctggcattatgc
ccagtacatgaccttatgggactttcctacttggcagtacatctacgtattagtcatcgctattaccatggtcgaggtgagccccacgttctgctt
cactctccccatctcccccccctccccacccccaattttgtatttatttattttttaattattttgtgcagcgatgggggcgggggggggggggg
ggcgcgcgccaggcggggcggggcggggcgaggggcggggcggggcgaggcggagaggtgcggcggcagccaatcagagcgg
cgcgctccgaaagtttccttttatggcgaggcggcggcggcggcggccctataaaaagcgaagcgcgcggcgggcgggagtcgctgcg
acgctgccttcgccccgtgccccgctccgccgccgcctcgcgccgcccgccccggctctgactgaccgcgttactcccacaggtgagcg
ggcgggacggcccttctcctccgggctgtaattagcgcttggtttaatgacggcttgtttcttttctgtggctgcgtgaaagccttgaggggctc
cgggagggccctttgtgcggggggagcggctcggggctgtccgcggggggacggctgccttcgggggggacggggcagggcgggg
ttcggcttctggcgtgtgaccggcggctctagagcctctgctaaccatgttcatgccttcttctttttcctacagctcctgggcaacgtgctggtt
attgtgctgtctcatcattttggcaaagaattggatcgggatccccgggtaCCGGTCGCCACCATGGTGAGCAAGGG
CGAGGAGCTGTTCACCGGGGTGGTGCCCATCCTGGTCGAGCTGGACGGCGACGTAA
ACGGCCACAAGTTCAGCGTGTCCGGCGAGGGCGAGGGCGATGCCACCTACGGCAAG
CTGACCCTGAAGTTCATCTGCACCACCGGCAAGCTGCCCGTGCCCTGGCCCACCCTC
GTGACCACCCTGACCTACGGCGTGCAGTGCTTCAGCCGCTACCCCGACCACATGAAG
CAGCACGACTTCTTCAAGTCCGCCATGCCCGAAGGCTACGTCCAGGAGCGCACCATC
TTCTTCAAGGACGACGGCAACTACAAGACCCGCGCCGAGGTGAAGTTCGAGGGCGA
CACCCTGGTGAACCGCATCGAGCTGAAGGGCATCGACTTCAAGGAGGACGGCAACA
TCCTGGGGCACAAGCTGGAGTACAACTACAACAGCCACAACGTCTATATCATGGCC
GACAAGCAGAAGAACGGCATCAAGGTGAACTTCAAGATCCGCCACAACATCGAGG
ACGGCAGCGTGCAGCTCGCCGACCACTACCAGCAGAACACCCCCATCGGCGACGGC
CCCGTGCTGCTGCCCGACAACCACTACCTGAGCACCCAGTCCGCCCTGAGCAAAGA
CCCCAACGAGAAGCGCGATCACATGGTCCTGCTGGAGTTCGTGACCGCCGCCGGGA
TCACTCTCGGCATGGACGAGctgtataagggtaaaaagaagaaaaagaagtcaaagacaaagtgtgtaattatgtagT
GTACAAGTCCGGACTCAGATCTCGAGCTCAagcttatcgataatcaacctctggattacaaaatttgtgaaagatt
gactggtattcttaactatgttgctccttttacgctatgtggatacgctgctttaatgcctttgtatcatgctattgcttcccgtatggctttcattttctc
ctccttgtataaatcctggttgctgtctctttatgaggagttgtggcccgttgtcaggcaacgtggcgtggtgtgcactgtgtttgctgacgcaac
ccccactggttggggcattgccaccacctgtcagctcctttccgggactttcgctttccccctccctattgccacggcggaactcatcgccgc
ctgccttgcccgctgctggacaggggctcggctgttgggcactgacaattccgtggtgttgtcggggaaatcatcgtcctttccttggctgctc
gcctgtgttgccacctggattctgcgcgggacgtccttctgctacgtcccttcggccctcaatccagcggaccttccttcccgcggcctgctg
ccggctctgcggcctcttccgcgtcttcgccttcgccctcagacgagtcggatctccctttgggccgcctccccgcatcgataccgtcgacc

cgggcggccgcttcgagcagacatgataagatacattgatgagtttggacaaaccacaactagaatgcagtgaaaaaaatgctttatttgtga
aatttgtgatgctattgctttatttgtaaccattataagctgcaataaacaagttaacaacaacaattgcattcattttatgtttcaggttcaggggga
gatgtgggaggttttttaaagcaagtaaaacctctacaaatgtggtaaaatcgataaggatcttcctagagcatggctacgtagataagtagca
tggcgggttaatcattaactacaaggaacccctagtgatggagttggccactccctctctgcgcgctcgctcgctcactgaggccgggcgac
caaaggtcgcccgacgcccgggctttgcccgggcggcctcagtgagcgagcgagcgcgcagccttaattaacctaattcactggccgtc
gttttacaacgtcgtgactgggaaaaccctggcgttacccaacttaatcgccttgcagcacatccccctttcgccagctggcgtaatagcgaa
gaggcccgcaccgatcgcccttcccaacagttgcgcagcctgaatggcgaatgggacgcgccctgtagcggcgcattaagcgcggcgg
gtgtggtggttacgcgcagcgtgaccgctacacttgccagcgccctagcgcccgctcctttcgctttcttcccttcctttctcgccacgttcgcc
ggctttccccgtcaagctctaaatcgggggctccctttagggttccgatttagtgctttacggcacctcgaccccaaaaaacttgattagggtg
atggttcacgtagtgggccatcgccctgatagacggtttttcgccctttgacgttggagtccacgttctttaatagtggactcttgttccaaactg
gaacaacactcaaccctatctcggtctattcttttgatttataagggattttgccgatttcggcctattggttaaaaaatgagctgatttaacaaaaa
tttaacgcgaattttaacaaaatattaacgcttacaatttaggtggcacttttcggggaaatgtgcgcggaacccctatttgtttatttttctaaatac
attcaaatatgtatccgctcatgagacaataaccctgataaatgcttcaataatattgaaaaaggaagagtatgagtattcaacatttccgtgtcg
cccttattcccttttttgcggcattttgccttcctgtttttgctcacccagaaacgctggtgaaagtaaaagatgctgaagatcagttgggtgcacg
agtgggttacatcgaactggatctcaacagcggtaagatccttgagagttttcgccccgaagaacgttttccaatgatgagcacttttaaagttc
tgctatgtggcgcggtattatcccgtattgacgccgggcaagagcaactcggtcgccgcatacactattctcagaatgacttggttgagtactc
accagtcacagaaaagcatcttacggatggcatgacagtaagagaattatgcagtgctgccataaccatgagtgataacactgcggccaac
ttacttctgacaacgatcggaggaccgaaggagctaaccgcttttttgcacaacatgggggatcatgtaactcgccttgatcgttgggaaccg
gagctgaatgaagccataccaaacgacgagcgtgacaccacgatgcctgtagcaatggcaacaacgttgcgcaaactattaactggcgaa
ctacttactctagcttcccggcaacaattaatagactggatggaggcggataaagttgcaggaccacttctgcgctcggcccttccggctggc
tggtttattgctgataaatctggagccggtgagcgtgggtctcgcggtatcattgcagcactggggccagatggtaagccctcccgtatcgta
gttatctacacgacggggagtcaggcaactatggatgaacgaaatagacagatcgctgagataggtgcctcactgattaagcattggtaact
gtcagaccaagtttactcatatatactttagattgatttaaaacttcatttttaatttaaaaggatctaggtgaagatcctttttgataatctcatgacc
aaaatcccttaacgtgagttttcgttccactgagcgtcagaccccgtagaaaagatcaaaggatcttc

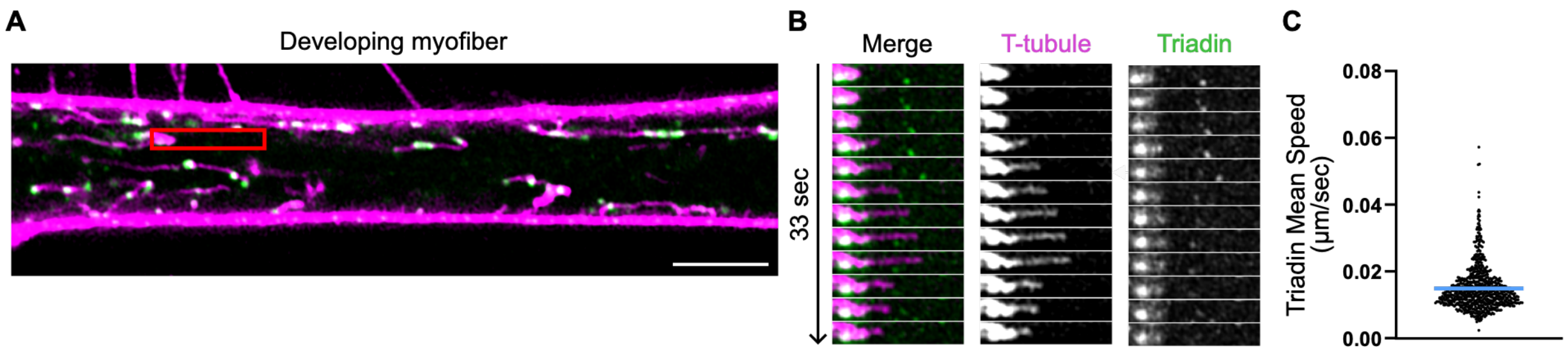

Fig. S1. T-tubules are dynamic independently of contacts with the ER.

Triadin-GFP, an ER transmembrane protein localizes at t-tubule - ER contact sites and triads (*81*). (**A**) Timelapse snapshot of an *in vitro* developing myofiber (differentiation day 4) expressing Triadin-GFP (green), and CellMask DeepRed (magenta) to label t-tubules, was imaged by timelapse every 3 s for 2 min. Scale bar, 5 µm. (**B**) Kymograph from red ROI showing that Triadin-GFP is static when t-tubules are growing. In 100% of growing t-tubules, we did not observe Triadin-GFP moving with the t-tubule (N=39 growing t-tubules). (**C**) The plot represents Triadin-GFP mean speed, calculated using trackmate, in five different cells from two independent repeats (N=783 tracks). Triadin-GFP exhibits a negligible average speed of 0.015 µm/s compared to the t-tubule growing average speed of 1.2 µm/s (Fig. 1B). Blue line on plot represents mean value.

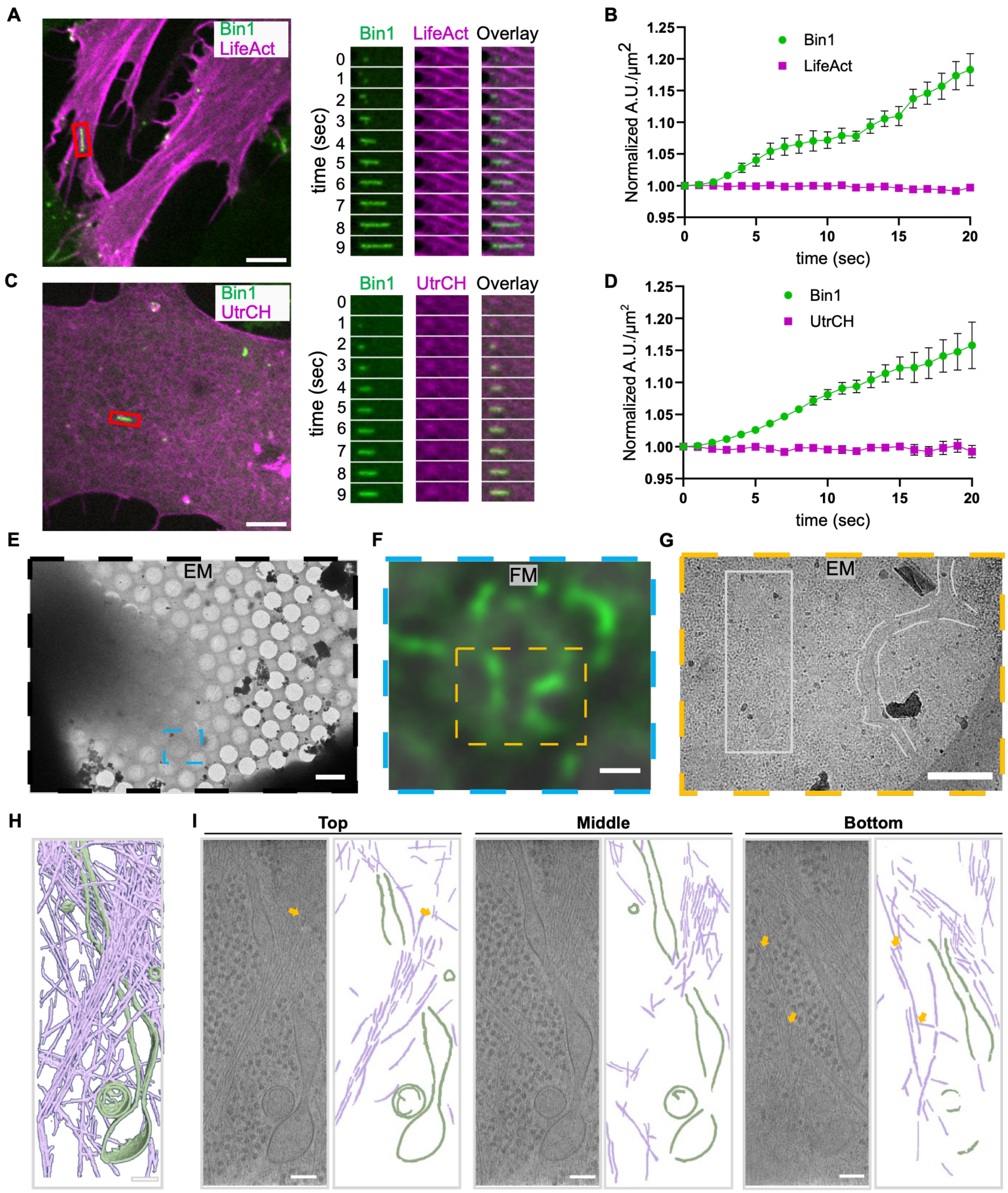

Fig. S2. F-actin is not locally nucleated at growing tubules.

(A) Left image corresponds to a timelapse snapshot of GFP-Bin1 myoblasts (green) and LifeAct-mCherry (magenta). Right image corresponds to kymographs from the red rectangle ROI showing a Bin1-tubule growing over time in the absence of F-actin changes. (**B**) Plots of LifeAct-mCherry and GFP-Bin1 average fluorescence intensity over time. Mean and SEM are represented. N=19 from two independent experiments. (**C**) Left image corresponds to a timelapse snapshot of GFP-Bin1 myoblasts (green) and mScarlet-I-UtrCH (magenta). Right image corresponds to kymographs from the red rectangle ROI showing a tubule growing over time in the absence of F-actin changes. (**D**) Plots of the mScarlet-I-UtrCH and GFP-Bin1 average fluorescence intensity over time. Mean and SEM are represented. N=16 from two independent experiments.

(**E**) Representative low magnification cryo-EM image showing part of one cell on a TEM grid. The cyan rectangle indicates the ROI imaged using epifluorescence and brightfield microscopy (panel F). (**F**) Merged epifluorescence (green) and brightfield (grey, FM) image of the ROI in E, showing multiple GFP-Bin1 tubules at the cell periphery. The orange rectangle indicates the ROI imaged using TEM (panel G). (**G**) Medium magnification TEM image of the ROI in F, showing the tubules observed in F. (**H**) 3D representation of cropped tomogram segmentation of the white rectangle ROI from G, with actin filaments (purple) and tubule membrane (green). (**I**) Slices of the tomogram in H at different z positions (from left to right: z=-25nm, 30nm and 135nm relative to the central slice) with actin filaments (purple), tubule membrane (green), and potential actin branches (yellow arrows). Scale bar: 5 µm in A, C and E, 1 µm in F, 500 nm in G and 100 nm in H and I.

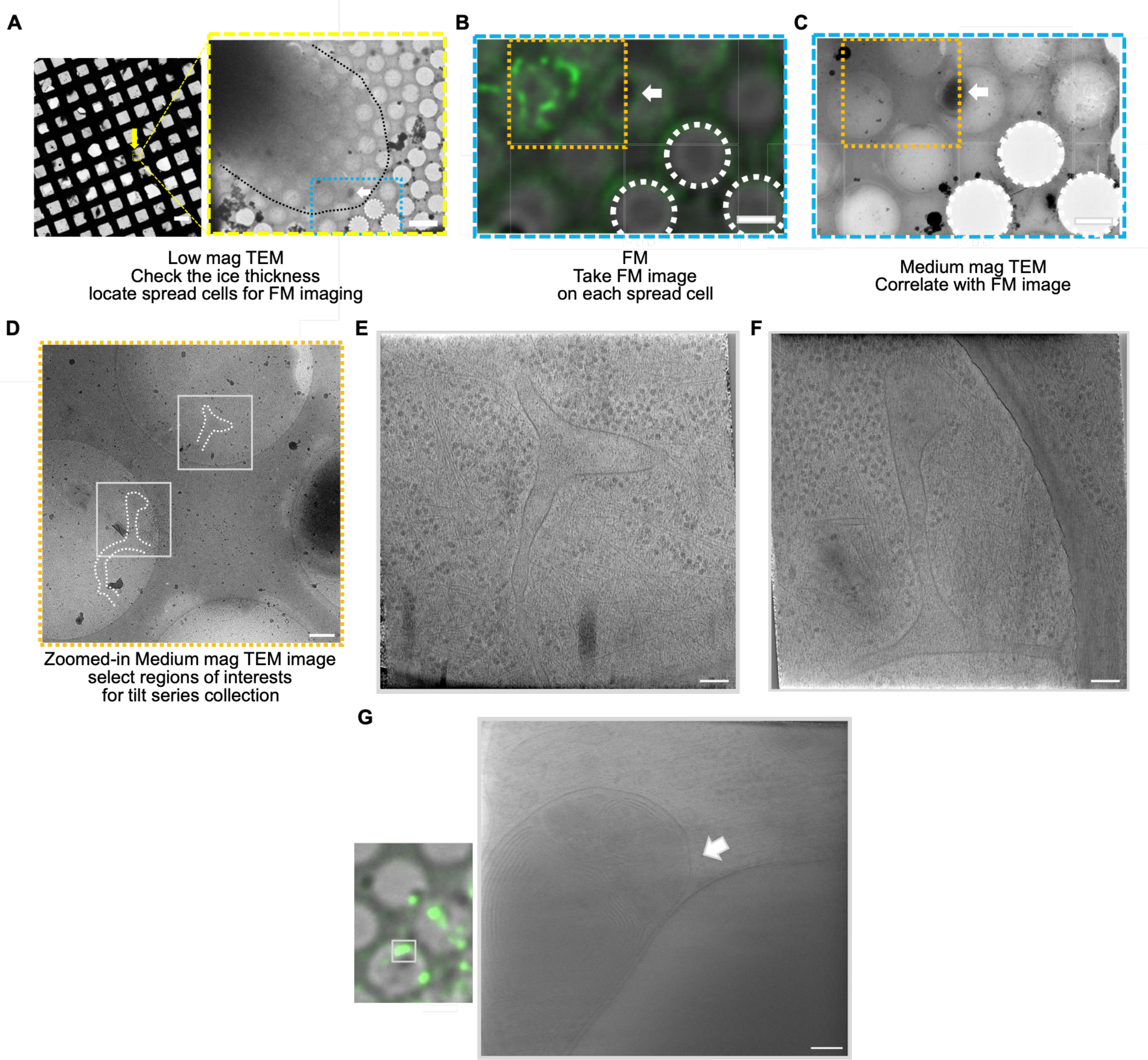

Fig. S3. Correlative light and electron microscopy workflow.

(**A**) Cells were imaged using TEM to check the ice thickness and locate spread cells for FM imaging. In the example image, one spread cell is indicated with a yellow arrow, with the approximate cell periphery outlined in black in the zoomed view. The image on the right is also shown in fig. S2E, and it is intentionally reused to illustrate the imaging workflow. (**B**) Cells imaged using cryo-fluorescence microscopy (FM) with the bright-field image of the area in the blue box from A showing the GFP-Bin1-labeled membrane tubule structures at the cell periphery. The image is also shown in fig. S2F, and it is intentionally reused to showcase the imaging workflow. (**C**) Cells were re-imaged using medium magnification TEM images in tiles and combined for FM-TEM correlation. The empty holes and black features were used for manual correlation, with empty holes indicated by white circles and the dark feature marked by a white arrow. (**D**) A zoomed-in view of the medium magnification image collected from the orange region in C. Tubular membrane structures are highlighted with white curvy lines. The shapes of these tubular membrane structures resemble those seen in the FM image in B. Regions of interest (ROI) were selected for tilt series data collection, indicated by white boxes. Part of the image is also

shown in fig. S2G, and it is intentionally reused to showcase the imaging workflow. (**E**) and (**F**) The central slice along the z-axis of the 3D tomogram at positions 1 and 2, respectively. (**G**) The bright-field and FM image showing fluorescently-labelled vesicular structure. A tomographic slice of the reconstruction inside the white box showing nested sets of membranes is shown on the right. Scale bar: 100 µm and 5 µm in A, 2 µm in B and C, 500 nm in D, 100 nm in E and F. 2 µm and 100 nm in G.

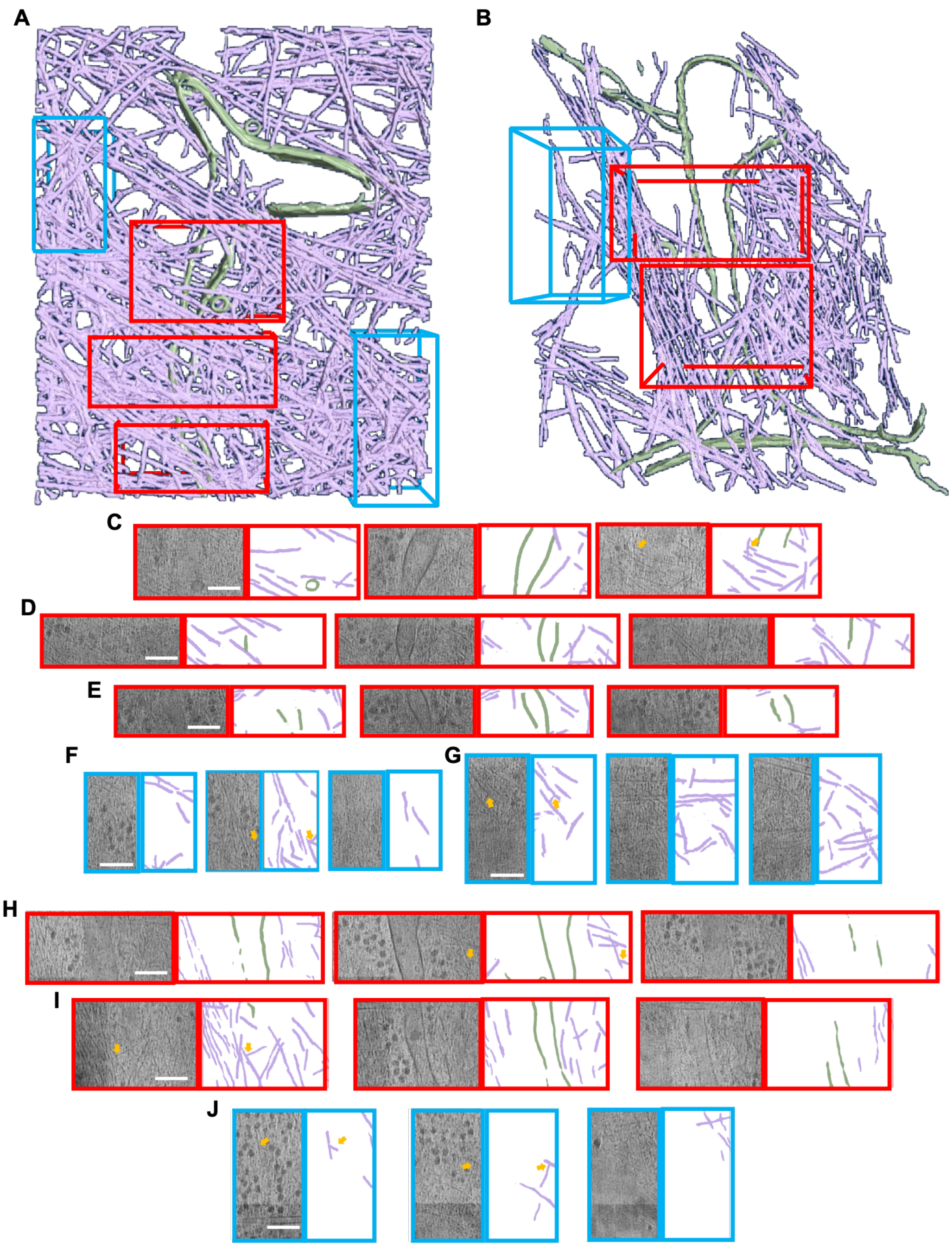

Fig. S4. Quantitative analysis of cryo-electron tomograms.

(**A**) 3D representation of cropped tomogram segmentation in fig. S3E, sub-volumes proximal (red ROI) or distant (blue ROI) from tubule membrane used for counting branch number indicated with red boxes and blue boxes, respectively. (**B**) 3D representation of cropped tomogram segmentation in fig. S3F, sub-volumes proximal to and distant from tubule membrane used for counting branch number indicated with red boxes and blue boxes, respectively. (**C**) Slices and segmentations of the tomogram in A in red box1 at different z positions (from left to right: z=-35nm, 5nm and 34nm relative to the central slice) with actin filaments in purple, tubules in green, and potential actin

branches indicated with yellow arrows. Scale bar, 100 nm. (**D**) Slices and segmentations of the tomogram in A in red box2 at different z positions (from left to right: z=-26nm, 3nm and 28nm relative to the central slice) with actin filaments in purple, tubules in green. Scale bar, 100 nm. (**E**) Slices and segmentations of the tomogram in A in red box3 at different z positions (from left to right: z=1 nm, 14 nm and 34nm relative to the central slice) with actin filaments in purple, tubules in green. Scale bar, 100 nm. (**F**) Slices and segmentations of the tomogram in A blue box1 at different z positions (from left to right: z=-27 nm, 3 nm and 22 nm relative to the central slice) with actin filaments in purple, tubules in green, and potential actin branches indicated with yellow arrows. Scale bar, 100 nm. (**G**) Slices and segmentations of the tomogram in A in blue box2 at different z positions (from left to right: z=-13nm, 10nm and 31nm relative to the central slice) with actin filaments in purple, tubules in green, and potential actin branches indicated with yellow arrows. Scale bar, 100 nm. (**H**) Slices and segmentations of the tomogram in B in red box1 at different z positions (from left to right: z=-15nm, 11nm and 34nm relative to the central slice) with actin filaments in purple, tubules in green, and potential actin branches indicated with yellow arrows. Scale bar, 100 nm. (**I**) Slices and segmentations of the tomogram in B in red box2 at different z positions (from left to right: z=-22 nm, 7 nm and 30 nm relative to the central slice) with actin filaments in purple, tubules in green, and potential actin branches indicated with yellow arrows. Scale bar, 100 nm. (**J**) Slices and segmentations of the tomogram in B in blue box at different z positions (from left to right: z=-14 nm, 16 nm and 39 nm relative to the central slice) with actin filaments in purple, tubules in green, and potential actin branches indicated with yellow arrows. Scale bar, 100 nm.

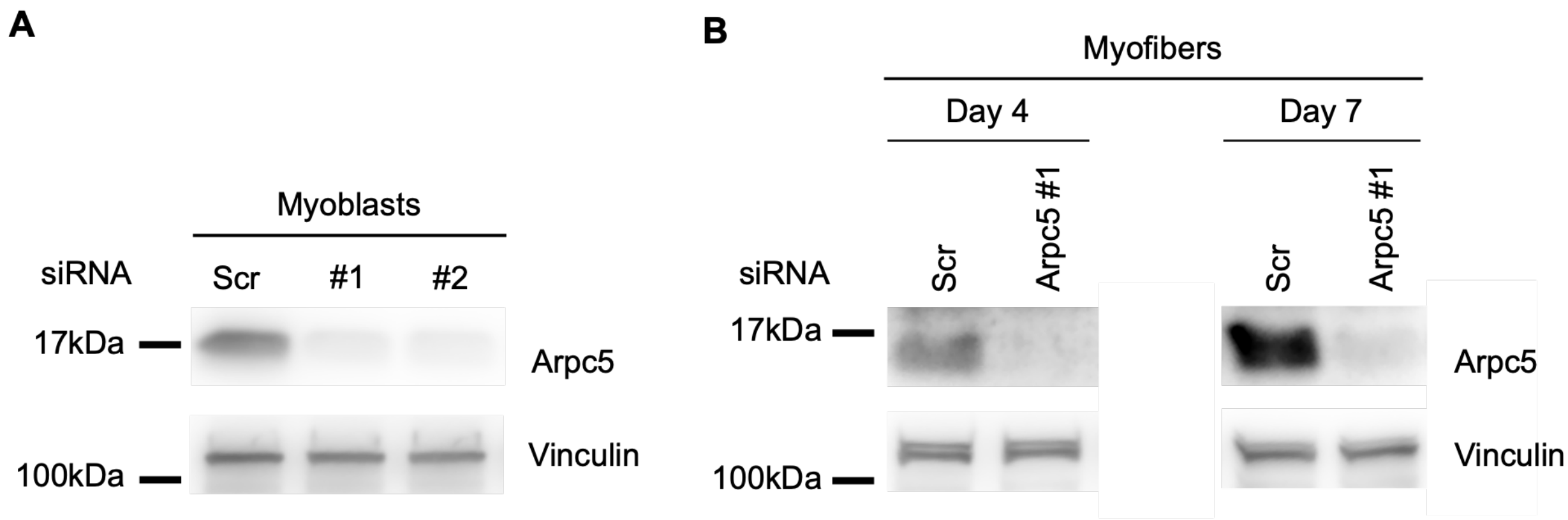

Fig. S5. Western blots of Arpc5 depletion.

(**A**) Western blots of GFP-Bin1 myoblasts knocked down for scramble (Scr), Arpc5 #1 (ID#s85623) or Arpc5 #2 (ID#s85621). Membranes were blotted using antibodies against Arpc5 with vinculin as a loading control. (**B**) Western blots of myofibers (differentiation day 4 or 7), knocked down for scramble (Scr) or Arpc5 #1. Membranes were blotted using antibodies against Arpc5 and vinculin, used as a loading control.

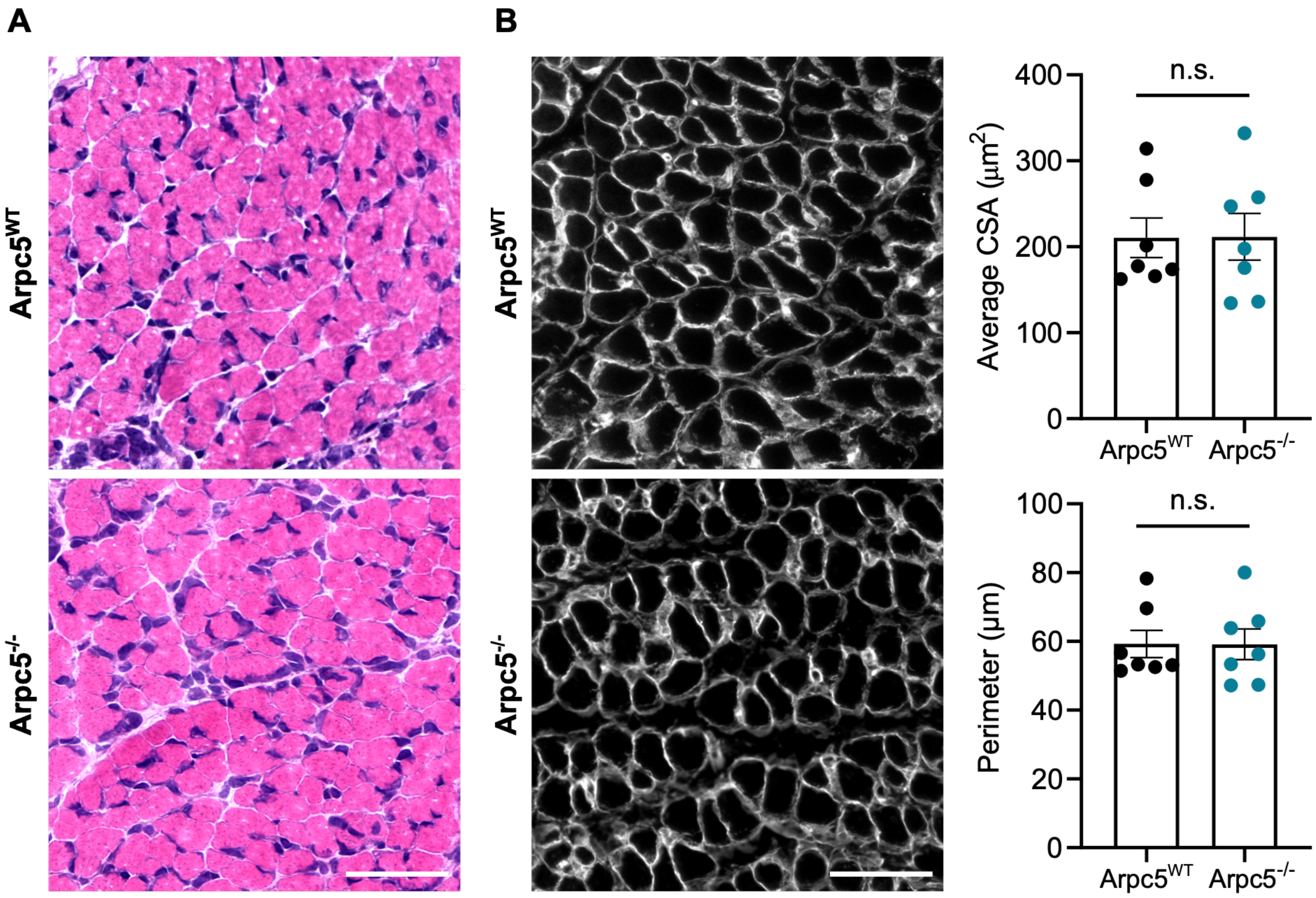

Fig. S6. Postnatal knockout of Arpc5 does not affect skeletal muscle morphology and size. (**A**) Representative images of cross-sectioned hindlimb muscle from P7 Arpc5^{WT} and Arpc5$^{-/-}$ mice, stained for Hematoxylin and Eosin (H&E). Scale bar, 50 µm. (**B**) (Left) Representative images of Arpc5^{WT} and Arpc5$^{-/-}$ TA muscle cross-sections stained for basal lamina (laminin, white). Scale bar, 50 µm. Quantifications of the mean myofiber CSA (right upper) and perimeter (right lower) in P7 Arpc5^{WT} and Arpc5$^{-/-}$ mice (N=7). Data are presented as mean ± s.e.m. n.s.= not significant using unpaired 2-tailed Student's t-test with Welch's correction or Mann-Whitney test based on normal distribution.

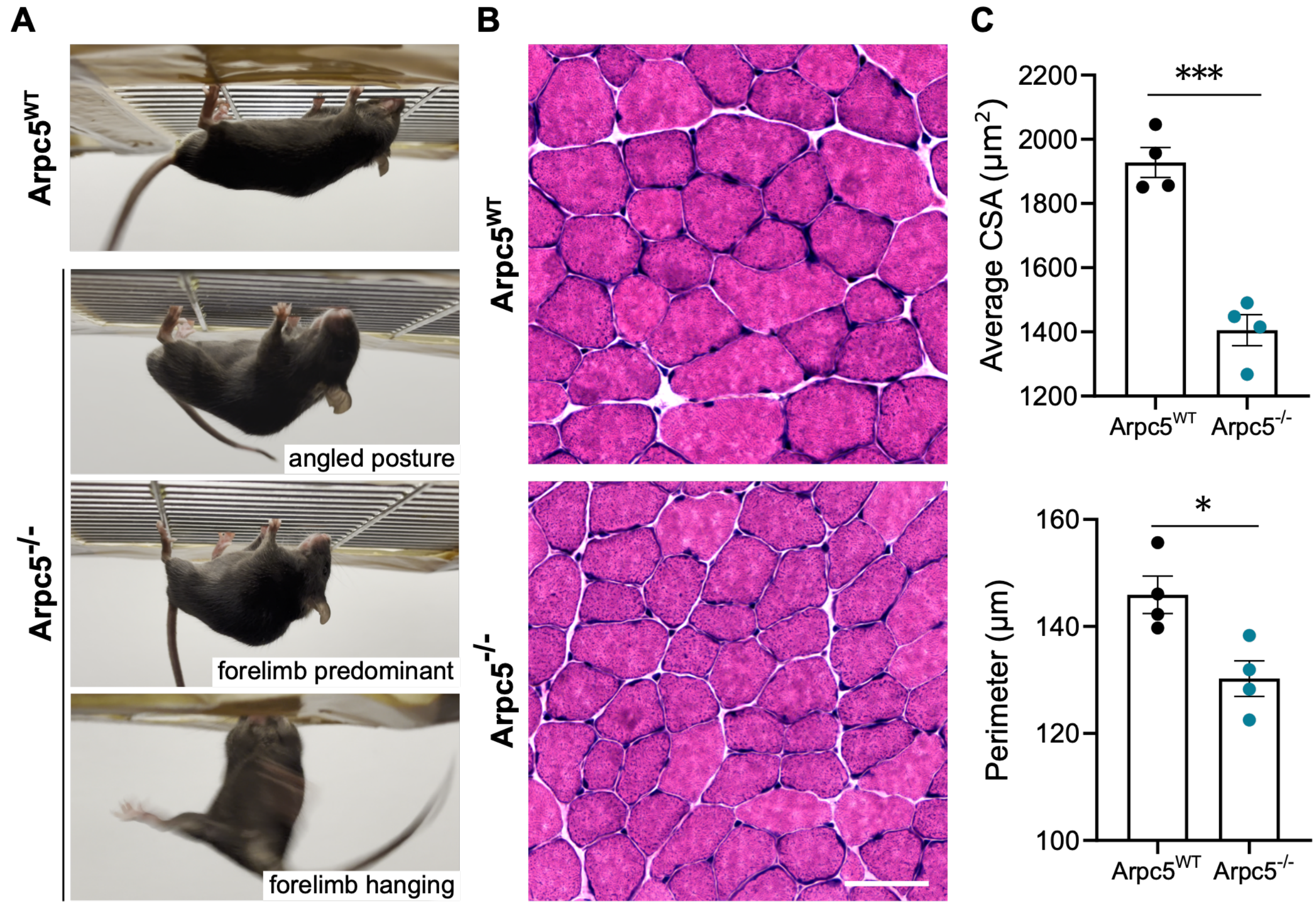

Fig. S7. Prolonged knockout of Arpc5 affects skeletal muscle strength and size.

(**A**) Representative frames of Arpc5^{WT} and Arpc5$^{-/-}$ mice performing the wire hanging test. Arpc5^{WT} animals maintain a stable grip with all four limbs keeping the body close to and parallel to the grid. Arpc5$^{-/-}$ animals adopted a more angled posture remaining farther from the grid (angled posture) or predominantly supported with forelimbs (forelimb predominant). In some cases, this resulted in complete loss of hindlimb grip and suspension only by forelimbs (forelimb hanging). (**B**) Representative images of cross-sectioned TA muscle from Arpc5^{WT} and Arpc5$^{-/-}$ mice, stained for Hematoxylin and Eosin (H&E). Scale bar, 50 µm. (**C**) Quantifications of the mean myofiber CSA (upper) and perimeter (lower) in TA cross-sections stained for the basal lamina (laminin, white in Fig. 6A) from adult Arpc5^{WT} and Arpc5$^{-/-}$ mice (N=4). Data are presented as mean ± s.e.m. *$p \leq 0.05$, ***$p \leq 0.001$ using unpaired 2-tailed Student's t-test with Welch's correction or Mann-Whitney test based on normal distribution.

Movie S1.

Timelapse movie of t-tubule dynamics in a myofiber isolated from the EDL muscle of an adult mouse. Isolated fiber was stained with CellMask to label t-tubules. Scale bar, 5 µm.

Movie S2.

Timelapse movie of t-tubule dynamics in a developing in vitro myofiber. Cells were stained with CellMask to label t-tubules. Scale bar, 5 µm.

Movie S3.

Timelapse movie of Triadin (green) and t-tubule (magenta) dynamics in a developing in vitro myofiber. Cells expressing Triadin-GFP are stained with CellMask to label t-tubules. Scale bar, 5 µm.

Movie S4.

Timelapse movie of t-tubule dynamics in a developing myofiber before (left panel) or 5 min after (right panel) adding CK666. Cells were stained with CellMask to label t-tubules. Scale bar, 5 µm.

Movie S5.

Timelapse movie of tubule dynamics in GFP-Bin1 myoblasts before and after adding CK666. Scale bar, 5 µm.

Movie S6.

F-actin (LifeAct-mCherry, magenta) and Bin1 tubules (GFP-Bin1, green) in GFP-Bin1 myoblasts. Scale bar, 5 µm.

Movie S7.

F-actin (mScarlet-I-UtrCH, magenta) and Bin1 tubules (GFP-Bin1, green) in GFP-Bin1 myoblasts. Scale bar, 5 µm.

Movie S8.

Cryo-electron tomogram and segmentation model of actin cytoskeleton around t-tubule site. Actin cytoskeleton coloured in light purple. T-tubule membrane coloured in green.

Movie S9.

Cryo-electron tomogram and segmentation model of actin cytoskeleton around t-tubule site. Actin cytoskeleton coloured in light purple. T-tubule membrane coloured in green.

Movie S10.

Cryo-electron tomogram and segmentation model of actin cytoskeleton around t-tubule site. Actin cytoskeleton coloured in light purple. T-tubule membrane coloured in green.

Movie S11.

Timelapse movie of tubule dynamics in GFP-Bin1 myoblasts before and after adding para-aminoblebbistatin (+Blebb). Scale bar, 5 µm.

Movie S12.

Timelapse movie of t-tubule dynamics in a developing in vitro myofiber before (left panel) or 5 min after (right panel) adding para-aminoblebbistatin (+Blebb). Cells were stained with CellMask to label t-tubules. Scale bar, 5 μm.

Movie S13.

Timelapse movie of tubule dynamics in GFP-Bin1 myoblasts before and after adding glycerol. Scale bar, 5 μm.

Movie S14.

Timelapse movie of t-tubule dynamics in a developing myofiber before (left panel) or after (right panel) adding glycerol. Cells were stained with CellMask to label t-tubules. Scale bar, 5 μm.

Movie S15.

Timelapse movie of tubule dynamics in GFP-Bin1 myoblasts before and after adding NSC668394 (+NSC). Scale bar, 5 μm.

Movie S16.

Timelapse movie of t-tubule dynamics in a developing in vitro myofiber before (left panel) or 5 min after (right panel) adding NSC668394 (+NSC). Cells were stained with CellMask to label t-tubules. Scale bar, 5 μm.

Movie S17.

Timelapse movie of tubule dynamics in GFP-Bin1 myoblasts depleted for scramble control (left panel) or depleted for Arpc5 siRNA#1. Scale bar, 5 μm.

Movie S18.

Timelapse movie of t-tubule dynamics in a developing myofiber in scramble cells (top panel) or in cells depleted for Arpc5 (bottom panel). T-tubules were stained with CellMask. Scale bar, 5 μm.

Movie S19.

MouseWalker tracked representative video (color-coded) of $Arpc5^{WT}$ (WT) and $Arpc5^{-/-}$ (Arpc5 KO) mice while walking.

REFERENCES

1. J. J. Dowling, C. C. Weihl, M. J. Spencer, Molecular and cellular basis of genetically inherited skeletal muscle disorders. *Nat. Rev. Mol. Cell Biol.* **22**, 713–732 (2021).

2. C. Franzini-Armstrong, The relationship between form and function throughout the history of excitation-contraction coupling. *J. Gen. Physiol.* **150**, 189–210 (2018).

3. L. Al-Qusairi, J. Laporte, T-tubule biogenesis and triad formation in skeletal muscle and implication in human diseases. *Skelet. Muscle* **1**, 26 (2011).

4. K. Kawaguchi, N. Fujita, Shaping transverse-tubules: Central mechanisms that play a role in the cytosol zoning for muscle contraction. *J. Biochem. (Tokyo)* **175**, 125–131 (2024).

5. K. R. Porter, G. E. Palade, Studies on the endoplasmic reticulum. III. Its form and distribution in striated muscle cells. *J. Biophys. Biochem. Cytol.* **3**, 269–300 (1957).

6. J. J. Dowling, M. W. Lawlor, R. T. Dirksen, Triadopathies: An emerging class of skeletal muscle diseases. *Neurother. J. Am. Soc. Exp. Neurother.* **11**, 773–785 (2014).

7. H. Takekura, B. E. Flucher, C. Franzini-Armstrong, Sequential docking, molecular differentiation, and positioning of T-Tubule/SR junctions in developing mouse skeletal muscle. *Dev. Biol.* **239**, 204–214 (2001).

8. E. Lemerle, J. Lainé, M. Benoist, G. Moulay, A. Bigot, C. Labasse, A. Madelaine, A. Canette, P. Aubin, J.-M. Vallat, N. B. Romero, M. Bitoun, V. Mouly, I. Marty, B. Cadot, L. Picas, S. Vassilopoulos, Caveolae and Bin1 form ring-shaped platforms for T-tubule initiation. *eLife* **12**, e84139 (2023).

9. T. E. Hall, N. Martel, N. Ariotti, Z. Xiong, H. P. Lo, C. Ferguson, J. Rae, Y.-W. Lim, R. G. Parton, In vivo cell biological screening identifies an endocytic capture mechanism for T-tubule formation. *Nat. Commun.* **11**, 3711 (2020).

10. L. D. Peachey, The sarcoplasmic reticulum and transverse tubules of the frog's sartorius. *J. Cell Biol.* **25**, 209–231 (1965).

11. P. Chugh, E. K. Paluch, The actin cortex at a glance. *J. Cell Sci.* **131**, jcs186254 (2018).

12. A. Jawahar, J. Vermeil, J. Heuvingh, O. du Roure, M. Piel, The third dimension of the actin cortex. *Curr. Opin. Cell Biol.* **89**, 102381 (2024).

13. T. M. Svitkina, Actin cell cortex: Structure and molecular organization. *Trends Cell Biol.* **30**, 556–565 (2020).

14. K. G. Campellone, N. M. Lebek, V. L. King, Branching out in different directions: Emerging cellular functions for the Arp2/3 complex and WASP-family actin nucleation factors. *Eur. J. Cell Biol.* **102**, 151301 (2023).

15. L. Cao, A. Yonis, M. Vaghela, E. H. Barriga, P. Chugh, M. B. Smith, J. Maufront, G. Lavoie, A. Méant, E. Ferber, M. Bovellan, A. Alberts, A. Bertin, R. Mayor, E. K. Paluch, P. P. Roux, A. Jégou, G. Romet-Lemonne, G. Charras, SPIN90 associates with mDia1 and the Arp2/3 complex to regulate cortical actin organization. *Nat. Cell Biol.* **22**, 803–814 (2020).

16. J. V. G. Abella, C. Galloni, J. Pernier, D. J. Barry, S. Kjær, M.-F. Carlier, M. Way, Isoform diversity in the Arp2/3 complex determines actin filament dynamics. *Nat. Cell Biol.* **18**, 76–86 (2016).

17. L. Cao, S. Huang, A. Basant, M. Mladenov, M. Way, CK-666 and CK-869 differentially inhibit Arp2/3 iso-complexes. *EMBO Rep.* **25**, 3221–3239 (2024).

18. C. Galloni, D. Carra, J. V. G. Abella, S. Kjær, P. Singaravelu, D. J. Barry, N. Kogata, C. Guérin, L. Blanchoin, M. Way, MICAL2 enhances branched actin network disassembly by oxidizing Arp3B-containing Arp2/3 complexes. *J. Cell Biol.* **220**, e202102043 (2021).

19. O. von Loeffelholz, A. Purkiss, L. Cao, S. Kjaer, N. Kogata, G. Romet-Lemonne, M. Way, C. A. Moores, Cryo-EM of human Arp2/3 complexes provides structural insights into actin nucleation modulation by ARPC5 isoforms. *Biol. Open* **9**, bio054304 (2020).

20. L. Cao, A. Basant, M. Mladenov, N. Kogata, A. Jegou, G. Romet-Lemonne, S. Brasselet, M. Mavrakis, M. Way, SPIN90 modulates the architecture of lamellipodial actin in an ARPC5L

dependent fashion. bioRxiv 691495 [Preprint] (2025). https://doi.org/10.64898/2025.12.01.691495.

21. F. Fäßler, M. G. Javoor, J. Datler, H. Döring, F. W. Hofer, G. Dimchev, V.-V. Hodirnau, J. Faix, K. Rottner, F. K. M. Schur, ArpC5 isoforms regulate Arp2/3 complex-dependent protrusion through differential Ena/VASP positioning. *Sci. Adv.* **9**, eadd6495 (2023).

22. L. Sadhu, N. Tsopoulidis, M. Hasanuzzaman, V. Laketa, M. Way, O. T. Fackler, ARPC5 isoforms and their regulation by calcium-calmodulin-N-WASP drive distinct Arp2/3-dependent actin remodeling events in CD4 T cells. *eLife* **12**, e82450 (2023).

23. W. Roman, J. P. Martins, F. A. Carvalho, R. Voituriez, J. V. G. Abella, N. C. Santos, B. Cadot, M. Way, E. R. Gomes, Myofibril contraction and crosslinking drive nuclear movement to the periphery of skeletal muscle. *Nat. Cell Biol.* **19**, 1189–1201 (2017).

24. E. Lee, M. Marcucci, L. Daniell, M. Pypaert, O. A. Weisz, G.-C. Ochoa, K. Farsad, M. R. Wenk, P. De Camilli, Amphiphysin 2 (Bin1) and T-tubule biogenesis in muscle. *Science* **297**, 1193–1196 (2002).

25. A.-S. Nicot, A. Toussaint, V. Tosch, C. Kretz, C. Wallgren-Pettersson, E. Iwarsson, H. Kingston, J.-M. Garnier, V. Biancalana, A. Oldfors, J.-L. Mandel, J. Laporte, Mutations in amphiphysin 2 (BIN1) disrupt interaction with dynamin 2 and cause autosomal recessive centronuclear myopathy. *Nat. Genet.* **39**, 1134–1139 (2007).

26. A. Allard, R. Lopes Dos Santos, C. Campillo, Remodelling of membrane tubules by the actin cytoskeleton. *Biol. Cell* **113**, 329–343 (2021).

27. A. Collins, A. Warrington, K. A. Taylor, T. Svitkina, Structural organization of the actin cytoskeleton at sites of clathrin-mediated endocytosis. *Curr. Biol. CB* **21**, 1167–1175 (2011).

28. D. Serwas, M. Akamatsu, A. Moayed, K. Vegesna, R. Vasan, J. M. Hill, J. Schöneberg, K. M. Davies, P. Rangamani, D. G. Drubin, Mechanistic insights into actin force generation during vesicle formation from cryo-electron tomography. *Dev. Cell* **57**, 1132–1145.e5 (2022).

29. M. Bovellan, Y. Romeo, M. Biro, A. Boden, P. Chugh, A. Yonis, M. Vaghela, M. Fritzsche, D. Moulding, R. Thorogate, A. Jégou, A. J. Thrasher, G. Romet-Lemonne, P. P. Roux, E. K. Paluch, G. Charras, Cellular control of cortical actin nucleation. *Curr. Biol.* **24**, 1628–1635 (2014).

30. M. Fritzsche, C. Erlenkämper, E. Moeendarbary, G. Charras, K. Kruse, Actin kinetics shapes cortical network structure and mechanics. *Sci. Adv.* **2**, e1501337 (2016).

31. V. Laplaud, N. Levernier, J. Pineau, M. S. Roman, L. Barbier, P. J. Sáez, A.-M. Lennon-Duménil, P. Vargas, K. Kruse, O. du Roure, M. Piel, J. Heuvingh, Pinching the cortex of live cells reveals thickness instabilities caused by myosin II motors. *Sci. Adv.* **7**, eabe3640 (2021).

32. B. H. Várkuti, M. Képiró, I. Á. Horváth, L. Végner, S. Ráti, Á. Zsigmond, G. Hegyi, Z. Lenkei, M. Varga, A. Málnási-Csizmadia, A highly soluble, non-phototoxic, non-fluorescent blebbistatin derivative. *Sci. Rep.* **6**, 26141 (2016).

33. C. Roffay, G. Molinard, K. Kim, M. Urbanska, V. Andrade, V. Barbarasa, P. Nowak, V. Mercier, J. García-Calvo, S. Matile, R. Loewith, A. Echard, J. Guck, M. Lenz, A. Roux, Passive coupling of membrane tension and cell volume during active response of cells to osmosis. *Proc. Natl. Acad. Sci. U.S.A.* **118**, e2103228118 (2021).

34. G. Bulut, S.-H. Hong, K. Chen, E. M. Beauchamp, S. Rahim, G. W. Kosturko, E. Glasgow, S. Dakshanamurthy, H.-S. Lee, I. Daar, J. A. Toretsky, C. Khanna, A. Uren, Small molecule inhibitors of ezrin inhibit the invasive phenotype of osteosarcoma cells. *Oncogene* **31**, 269–281 (2012).

35. M. Fritzsche, R. Thorogate, G. Charras, Quantitative analysis of ezrin turnover dynamics in the actin cortex. *Biophys. J.* **106**, 343–353 (2014).

36. S. Falcone, W. Roman, K. Hnia, V. Gache, N. Didier, J. Lainé, F. Auradé, I. Marty, I. Nishino, N. Charlet-Berguerand, N. B. Romero, G. Marazzi, D. Sassoon, J. Laporte, E. R. Gomes, N-WASP is required for Amphiphysin-2/BIN1-dependent nuclear positioning and triad

organization in skeletal muscle and is involved in the pathophysiology of centronuclear myopathy. *EMBO Mol. Med.* **6**, 1455–1475 (2014).

37. B. E. Flucher, S. B. Andrews, M. P. Daniels, Molecular organization of transverse tubule/sarcoplasmic reticulum junctions during development of excitation-contraction coupling in skeletal muscle. *Mol. Biol. Cell* **5**, 1105–1118 (1994).

38. C. Franzini-Armstrong, Simultaneous maturation of transverse tubules and sarcoplasmic reticulum during muscle differentiation in the mouse. *Dev. Biol.* **146**, 353–363 (1991).

39. E. Sindram, A. Caballero-Oteyza, N. Kogata, S. C. M. Huang, Z. Alizadeh, L. Gámez-Díaz, M. R. Fazlollhi, X. Peng, B. Grimbacher, M. Way, M. Proietti, ARPC5 deficiency leads to severe early-onset systemic inflammation and mortality. *Dis. Model. Mech.* **16**, dmm050145 (2023).

40. J. J. McCarthy, R. Srikuea, T. J. Kirby, C. A. Peterson, K. A. Esser, Inducible Cre transgenic mouse strain for skeletal muscle-specific gene targeting. *Skelet. Muscle* **2**, 8 (2012).

41. A. F. Isidro, A. M. Medeiros, I. Martins, D. Neves-Silva, L. Saúde, C. S. Mendes, Using the mousewalker to quantify locomotor dysfunction in a mouse model of spinal cord injury. *J. Vis. Exp.*, e65207 (2023).

42. C. S. Mendes, I. Bartos, Z. Márka, T. Akay, S. Márka, R. S. Mann, Quantification of gait parameters in freely walking rodents. *BMC Biol.* **13**, 50 (2015).

43. A. Toussaint, B. S. Cowling, K. Hnia, M. Mohr, A. Oldfors, Y. Schwab, U. Yis, T. Maisonobe, T. Stojkovic, C. Wallgren-Pettersson, V. Laugel, A. Echaniz-Laguna, J.-L. Mandel, I. Nishino, J. Laporte, Defects in amphiphysin 2 (BIN1) and triads in several forms of centronuclear myopathies. *Acta Neuropathol. (Berl.)* **121**, 253–266 (2011).

44. V. Guarani, C. Jardel, D. Chrétien, A. Lombès, P. Bénit, C. Labasse, E. Lacène, A. Bourillon, A. Imbard, J.-F. Benoist, I. Dorboz, M. Gilleron, E. S. Goetzman, P. Gaignard, A. Slama, M. Elmaleh-Bergès, N. B. Romero, P. Rustin, H. Ogier de Baulny, J. A. Paulo, J. W. Harper, M. Schiff, QIL1 mutation causes MICOS disassembly and early onset fatal mitochondrial encephalopathy with liver disease. *eLife* **5**, e17163 (2016).

45. F. Magri, S. Antognozzi, M. Ripolone, S. Zanotti, L. Napoli, P. Ciscato, D. Velardo, G. Scuvera, V. Nicotra, A. Giacobbe, D. Milani, F. Fortunato, M. Garbellini, M. Sciacco, S. Corti, G. P. Comi, D. Ronchi, Megaconial congenital muscular dystrophy due to novel CHKB variants: A case report and literature review. *Skelet. Muscle* **12**, 23 (2022).

46. A. J. Kosmalska, L. Casares, A. Elosegui-Artola, J. J. Thottacherry, R. Moreno-Vicente, V. González-Tarragó, M. Á. Del Pozo, S. Mayor, M. Arroyo, D. Navajas, X. Trepat, N. C. Gauthier, P. Roca-Cusachs, Physical principles of membrane remodelling during cell mechanoadaptation. *Nat. Commun.* **6**, 7292 (2015).

47. Y. Li, R. Lipowsky, R. Dimova, Membrane nanotubes induced by aqueous phase separation and stabilized by spontaneous curvature. *Proc. Natl. Acad. Sci. U.S.A.* **108**, 4731–4736 (2011).

48. L. Picas, J. Viaud, K. Schauer, S. Vanni, K. Hnia, V. Fraisier, A. Roux, P. Bassereau, F. Gaits-Iacovoni, B. Payrastre, J. Laporte, J.-B. Manneville, B. Goud, BIN1/M-Amphiphysin2 induces clustering of phosphoinositides to recruit its downstream partner dynamin. *Nat. Commun.* **5**, 5647 (2014).

49. V. M. Lionello, C. Kretz, E. Edelweiss, C. Crucifix, R. Gómez-Oca, N. Messaddeq, S. Buono, P. Koebel, X. Massana Muñoz, N. Diedhiou, B. S. Cowling, M. Bitoun, J. Laporte, BIN1 modulation in vivo rescues dynamin-related myopathy. *Proc. Natl. Acad. Sci. U.S.A.* **119**, e2109576119 (2022).

50. T. Hong, H. Yang, S.-S. Zhang, H. C. Cho, M. Kalashnikova, B. Sun, H. Zhang, A. Bhargava, M. Grabe, J. Olgin, J. Gorelik, E. Marbán, L. Y. Jan, R. M. Shaw, Cardiac BIN1 folds T-tubule membrane, controlling ion flux and limiting arrhythmia. *Nat. Med.* **20**, 624–632 (2014).

51. T. Hong, R. M. Shaw, Cardiac T-tubule microanatomy and function. *Physiol. Rev.* **97**, 227–252 (2017).

52. H. C. Spooner, R. E. Dixon, Bend it like BIN1: How a membrane-curving adaptor protein shapes cardiac physiology. *Am. J. Physiol.Heart Circ. Physiol.* **329**, H94–H108 (2025).

53. I. Jayasinghe, B. S. Launikonis, Three-dimensional reconstruction and analysis of the tubular system of vertebrate skeletal muscle. *J. Cell Sci.* **126**, 4048–4058 (2013).

54. S. Boncompagni, L. d'Amelio, S. Fulle, G. Fanò, F. Protasi, Progressive disorganization of the excitation–contraction coupling apparatus in aging human skeletal muscle as revealed by electron microscopy: A possible role in the decline of muscle performance. *J. Gerontol. Ser. A* **61**, 995–1008 (2006).

55. K. G. Claeys, T. Maisonobe, J. Böhm, J. Laporte, M. Hezode, N. B. Romero, G. Brochier, M. Bitoun, R. Y. Carlier, T. Stojkovic, Phenotype of a patient with recessive centronuclear myopathy and a novel BIN1 mutation. *Neurology* **74**, 519–521 (2010).

56. M. W. Lawlor, J. J. Dowling, X-linked myotubular myopathy. *Neuromuscul. Disord.* **31**, 1004–1012 (2021).

57. A. Y. Mejaddam, I. Nennesmo, T. Sejersen, Severe phenotype of a patient with autosomal recessive centronuclear myopathy due to a BIN1 mutation. *Acta Myol.* **28**, 91–93 (2009).

58. E. C. Oates, K. J. Jones, S. Donkervoort, A. Charlton, S. Brammah, J. E. Smith, J. S. Ware, K. S. Yau, L. C. Swanson, N. Whiffin, A. J. Peduto, A. Bournazos, L. B. Waddell, M. A. Farrar, H. A. Sampaio, H. L. Teoh, P. J. Lamont, D. Mowat, R. B. Fitzsimons, A. J. Corbett, M. M. Ryan, G. L. O'Grady, S. A. Sandaradura, R. Ghaoui, H. Joshi, J. L. Marshall, M. A. Nolan, S. Kaur, J. Punetha, A. Töpf, E. Harris, M. Bakshi, C. A. Genetti, M. Marttila, U. Werlauff, N. Streichenberger, A. Pestronk, I. Mazanti, J. R. Pinner, C. Vuillerot, C. Grosmann, A. Camacho, P. Mohassel, M. E. Leach, A. R. Foley, D. Bharucha-Goebel, J. Collins, A. M. Connolly, H. R. Gilbreath, S. T. Iannaccone, D. Castro, B. B. Cummings, R. I. Webster, L. Lazaro, J. Vissing, S. Coppens, N. Deconinck, H.-M. Luk, N. H. Thomas, N. C. Foulds, M. A. Illingworth, S. Ellard, C. A. McLean, R. Phadke, G. Ravenscroft, N. Witting, P. Hackman, I. Richard, S. T. Cooper, E.-J. Kamsteeg, E. P. Hoffman, K. Bushby, V. Straub, B. Udd, A. Ferreiro, K. N. North, N. F. Clarke, M. Lek, A. H. Beggs, C. G. Bönnemann, D. G. MacArthur, H. Granzier, M. R. Davis, N. G. Laing, Congenital titinopathy: Comprehensive characterization and pathogenic insights. *Ann. Neurol.* **83**, 1105–1124 (2018).

59. C. J. Nunes-Santos, H. Kuehn, B. Boast, S. Hwang, D. B. Kuhns, J. Stoddard, J. E. Niemela, D. L. Fink, S. Pittaluga, M. Abu-Asab, J. S. Davies, V. A. Barr, T. Kawai, O. M. Delmonte, M. Bosticardo, M. Garofalo, M. Carneiro-Sampaio, R. Somech, M. Gharagozlou, N. Parvaneh, L. E. Samelson, T. A. Fleisher, A. Puel, L. D. Notarangelo, B. Boisson, J.-L. Casanova, B. Derfalvi, S. D. Rosenzweig, Inherited ARPC5 mutations cause an actinopathy impairing cell motility and disrupting cytokine signaling. *Nat. Commun.* **14**, 3708 (2023).

60. M. R. Pimentel, S. Falcone, B. Cadot, E. R. Gomes, In vitro differentiation of mature myofibers for live imaging. *J. Vis. Exp.*, 55141 (2017).

61. D. N. Feather-Schussler, T. S. Ferguson, A battery of motor tests in a neonatal mouse model of cerebral palsy. *J. Vis. Exp.*, 53569 (2016).

62. S. M. Klein, J. Vykoukal, P. Lechler, K. Zeitler, S. Gehmert, S. Schreml, E. Alt, U. Bogdahn, L. Prantl, Noninvasive in vivo assessment of muscle impairment in the mdx mouse model—A comparison of two common wire hanging methods with two different results. *J. Neurosci. Methods* **203**, 292–297 (2012).

63. NeurogeneLocomotion, *Neurogene Locomotion/MouseWalker* (2024); https://github.com/NeurogeneLocomotion/MouseWalker.

64. C. S. Janota, A. Pinto, A. Pezzarossa, P. Machado, J. Costa, P. Campinho, C. A. Franco, E. R. Gomes, Shielding of actin by the endoplasmic reticulum impacts nuclear positioning. *Nat. Commun.* **13**, 2763 (2022).

65. A. Fourest-Lieuvin, J. Rendu, A. Osseni, K. Pernet-Gallay, D. Rossi, S. Oddoux, J. Brocard, V. Sorrentino, I. Marty, J. Fauré, Role of triadin in the organization of reticulum membrane at the muscle triad. *J. Cell Sci.* **125**, 3443–3453 (2012).

66. A. the nonprofit plasmid Repository, AAV Production in HEK293T Cells V.2. (2019); https:/protocols.io/view/aav-production-in-hek293t-cells-bawpifdn.pdf.

67. Addgene the nonprofit plasmid Repository, AAV Purification by Iodixanol Gradient Ultracentrifugation V.1. (2019); https://dx.doi.org/10.17504/protocols.io.47jgzkn.

68. J. Schindelin, I. Arganda-Carreras, E. Frise, V. Kaynig, M. Longair, T. Pietzsch, S. Preibisch, C. Rueden, S. Saalfeld, B. Schmid, J.-Y. Tinevez, D. J. White, V. Hartenstein, K. Eliceiri, P. Tomancak, A. Cardona, Fiji: An open-source platform for biological-image analysis. *Nat. Methods* **9**, 676–682 (2012).

69. T. Ferreira, K. Miura, B. Chef, J. Eglinger, *Scripts: BAR 1.1.6* (Zenodo, 2015); https://zenodo.org/records/28838.

70. J. W. Pylvänäinen, R. F. Laine, B. M. S. Saraiva, S. Ghimire, G. Follain, R. Henriques, G. Jacquemet, Fast4DReg—Fast registration of 4D microscopy datasets. *J. Cell Sci.* **136**, jcs260728 (2023).

71. D. Ershov, M.-S. Phan, J. W. Pylvänäinen, S. U. Rigaud, L. Le Blanc, A. Charles-Orszag, J. R. W. Conway, R. F. Laine, N. H. Roy, D. Bonazzi, G. Duménil, G. Jacquemet, J.-Y. Tinevez, TrackMate 7: Integrating state-of-the-art segmentation algorithms into tracking pipelines. *Nat. Methods* **19**, 829–832 (2022).

72. C. Stringer, T. Wang, M. Michaelos, M. Pachitariu, Cellpose: A generalist algorithm for cellular segmentation. *Nat. Methods* **18**, 100–106 (2021).

73. J. Zivanov, J. Otón, Z. Ke, A. von Kügelgen, E. Pyle, K. Qu, D. Morado, D. Castaño-Díez, G. Zanetti, T. A. M. Bharat, J. A. G. Briggs, S. H. W. Scheres, A Bayesian approach to single-particle electron cryo-tomography in RELION-4.0. *eLife* **11**, e83724 (2022).

74. S. Q. Zheng, E. Palovcak, J.-P. Armache, K. A. Verba, Y. Cheng, D. A. Agard, MotionCor2: Anisotropic correction of beam-induced motion for improved cryo-electron microscopy. *Nat. Methods* **14**, 331–332 (2017).

75. A. Rohou, N. Grigorieff, CTFFIND4: Fast and accurate defocus estimation from electron micrographs. *J. Struct. Biol.* **192**, 216–221 (2015).

76. T.-O. Buchholz, A. Krull, R. Shahidi, G. Pigino, G. Jékely, F. Jug, Content-aware image restoration for electron microscopy. *Methods Cell Biol.* **152**, 277–289 (2019).

77. Y.-T. Liu, H. Zhang, H. Wang, C.-L. Tao, G.-Q. Bi, Z. H. Zhou, Isotropic reconstruction for electron tomography with deep learning. *Nat. Commun.* **13**, 6482 (2022).

78. E. F. Pettersen, T. D. Goddard, C. C. Huang, E. C. Meng, G. S. Couch, T. I. Croll, J. H. Morris, T. E. Ferrin, UCSF ChimeraX: Structure visualization for researchers, educators, and developers. *Protein Sci. Publ. Protein Soc.* **30**, 70–82 (2021).

79. T.-W. Chen, T. J. Wardill, Y. Sun, S. R. Pulver, S. L. Renninger, A. Baohan, E. R. Schreiter, R. A. Kerr, M. B. Orger, V. Jayaraman, L. L. Looger, K. Svoboda, D. S. Kim, Ultrasensitive fluorescent proteins for imaging neuronal activity. *Nature* **499**, 295–300 (2013).

80. J. R. Kremer, D. N. Mastronarde, J. R. McIntosh, Computer visualization of three-dimensional image data using IMOD. *J. Struct. Biol.* **116**, 71–76 (1996).

81. M. Sébastien, P. Aubin, J. Brocard, J. Brocard, I. Marty, J. Fauré, Dynamics of triadin, a muscle-specific triad protein, within sarcoplasmic reticulum subdomains. *Mol. Biol. Cell* **31**, 261–272 (2020).
